# Characterization of Dopaminergic System in the Striatum of Young Adult *Park2*^−/−^ Knockout Rats

**DOI:** 10.1038/s41598-017-18526-0

**Published:** 2018-01-24

**Authors:** Jickssa M. Gemechu, Akhil Sharma, Dongyue Yu, Yuran Xie, Olivia M. Merkel, Anna Moszczynska

**Affiliations:** 1 Department of Pharmaceutical Sciences, Wayne State University, Detroit, MI USA; 2 Department of Biomedical Sciences, OUWB School of Medicine, Rochester, MI USA; 3 Department of Pharmaceutical Sciences, University of Maryland School of Pharmacy, Baltimore, MD USA; 4 Boston Biomedical Inc., Allston, MA USA; 5 Department of Pharmacy, Ludwig-Maximilians University of Munich, Munich, Germany

## Abstract

Mutations in parkin gene (*Park2*) are linked to early-onset autosomal recessive Parkinson’s disease (PD) and young-onset sporadic PD. *Park2* knockout (PKO) rodents; however, do not display neurodegeneration of the nigrostriatal pathway, suggesting age-dependent compensatory changes. Our goal was to examine dopaminergic (DAergic) system in the striatum of 2 month-old PKO rats in order to characterize compensatory mechanisms that may have occurred within the system. The striata form wild type (WT) and PKO Long Evans male rats were assessed for the levels of DAergic markers, for monoamine oxidase (MAO) A and B activities and levels, and for the levels of their respective preferred substrates, serotonin (5-HT) and ß-phenylethylamine (ß-PEA). The PKO rats displayed lower activities of MAOs and higher levels of ß-PEA in the striatum than their WT counterparts. Decreased levels of ß-PEA receptor, trace amine-associated receptor 1 (TAAR-1), and postsynaptic DA D2 (D2L) receptor accompanied these alterations. Drug-naive PKO rats displayed normal locomotor activity; however, they displayed decreased locomotor response to a low dose of psychostimulant methamphetamine, suggesting altered DAergic neurotransmission in the striatum when challenged with an indirect agonist. Altogether, our findings suggest that 2 month-old PKO male rats have altered DAergic and trace aminergic signaling.

## Introduction

Parkin is an E3 ubiquitin-protein ligase with neuroprotective properties. The main function of parkin is associated with the ubiquitin-proteasome system, a predominant cellular pathway responsible for targeting specific proteins for degradation. Parkin has also other functions e.g., regulation of trafficking of membrane proteins, including dopamine transporter (DAT), maintenance of mitochondrial homeostasis, and axonal transport^1^. Mutations in parkin gene (*Park2*) account for 50% of familial Parkinson’s disease (PD) cases, causing autosomal recessive juvenile PD, and for approximately 20% of young onset sporadic PD^2,3^. Oxidative stress and aggregation-mediated parkin loss of function is also linked to sporadic PD^4,5^. Conversely, overexpression of parkin protects dopamine (DA) neurons in animal models of the disease^6,7^, suggesting the importance of parkin in DA neuron function and maintenance.

Despite intensive research, the exact role of parkin deficit in development of PD is still unclear. Majority of parkin knockout (PKO) mouse models, or recently generated PKO rats, do not display PD-like motor impairments or any indication of progressive degeneration of nigrostriatal DA pathway^8,9^. Some of parkin mutation models, conditional parkin knockout, and combination of parkin gene knockout with a knockout of other gene were able to recapitulate major features of PD^8,10^. In majority of the aforementioned studies, PKO rodents were examined for nigrostriatal pathway degeneration at the later stages of life. The failure of older animals to develop PD could have been caused by compensatory mechanisms in the brain^11–14^. Younger PKO rodents may, at least in part, mimic pre-symptomatic stages of PD^15,16^. The studies in PKO mice suggest that loss of parkin protein may result in decreased release of presynaptic DA (reviewed in^8,10^).

The major objective of the present study was to characterize the DAergic system in the striatum of young adult (2 month-old) PKO rats in order to characterize compensatory mechanisms that may have occurred in the striatum lacking parkin as well as to elucidate potential early signs of PD in these rats. We measured the levels of DA and its metabolites, the levels of DA synthesizing enzyme tyrosine hydroxylase (TH), the levels of dopamine transporter (DAT), vesicular monoamine transporter 2 (VMAT2), and DA D2 receptor isoforms (D2S and D2L) in the striatum of male PKO and WT Long Evans rats. In addition, we assessed the activities and levels of DA metabolizing enzymes, monoamine oxidase A and B (MAO-A and MAO-B), and the levels of MAO-A and MAO-B preferred substrates, serotonin (5-HT) and ß-phenylethylamine (ß-PEA), respectively. Of relevance to the objective of this study, 5-HT and ß-PEA are known regulators DAergic neurotransmission^17,18^. We have also examined the expression of genes coding for proteins with altered levels. Finally, we assessed basal motor activity and locomotor response to a low dose of a psychostimulant methamphetamine in the PKO *vs*. WT rats.

The 2 month-old PKO rats displayed no signs DAergic neurotoxicity (i.e. deficits in DAergic markers) in the striatum. However, several alterations relevant to DAergic neurotransmission were detected. The activity of MAO-A and particularly the activity of MAO-B were significantly decreased. Despite these decreases, there was no indication of the presence of DA-mediated oxidative stress in the striatum of these rats as assessed by lack of an increase in the levels of DA quinones or lipid peroxidation by-product, 2-hydroxynonenal (4-HNE). The decreased activity of MAO-B was accompanied by significantly increased levels of MAO-B preferred substrate ß-PEA and decreased levels of trace amine-associated receptor 1 (TAAR-1), a receptor for ß-PEA. We also found decreased levels of striatal postsynaptic DA D2L receptor in the knockouts. The expression of *DRD2* gene was increased while the expression of *MAOB* and *TAAR1* genes was unaltered which led us to examine transactivation DNA-binding protein (TDP-43), a DNA/RNA-binding protein involved in regulation of transcription and translation processes, and a substrate of parkin. We found a decrease in the levels of TDP-43 protein. The basal locomotor activity was not significantly different between the genotypes, while rapid stereotypic movements were significantly but slightly decreased in the PKO rats. The locomotor response to methamphetamine was decreased in the knockouts compared to the WT controls.

Collectively, our results indicate that loss of parkin protein does not result in development of significant DAergic deficits or motor abnormalities in 2 month-old PKO rats; however, it leads to some alterations in DAergic and trace aminergic signaling aimed to balance the behavioral outcome. Environmental insults, such as methamphetamine abuse, might destroy this compensatory equilibrium and lead to development of Parkinsonian features. Our results strengthen the idea that species differences might have implications for the individualized treatment of PD.

## Results

### Validation of *Park2* gene knockout

**Figure 1:**
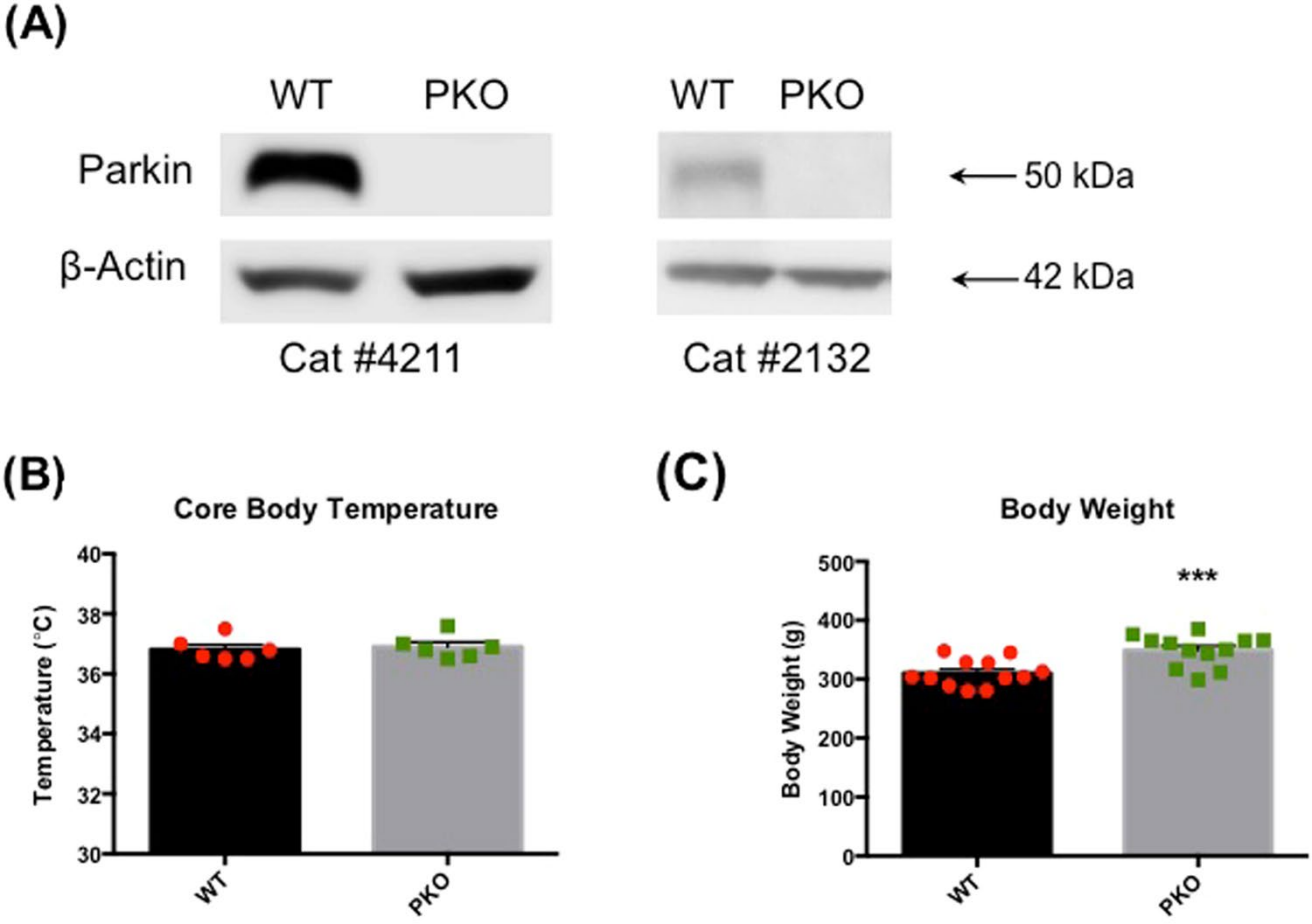
Validation of *Park2* gene knockout and the effects of parkin deficit on core body temperature and body weight. (**A**) Homozygous parkin knockout (PKO) and wild type (WT) Long Evans male rats were sacrificed at the age of 2 months. The striata were dissected out and analyzed for parkin immunoreactivity using SDS-PAGE and western blot analysis with two different anti-parkin antibodies. The analysis demonstrated a complete loss of parkin immunoreactivity in the PKO rats (n = 6). (**B**) Core body temperatures and body weight was assessed in the WT and PKO rats. The PKO rats displayed normal core body temperature (**B**) and higher body weight than the age-matched WT controls (+12.6%, Student’s t-test, p < 0.001, n = 12) (**C**). The data are expressed as mean ± SEM. ***p < 0.001. Abbreviations: PKO, parkin knockout; WT, wild type.

The loss of parkin protein in the homozygous PKO rats was ascertained using SDS-PAGE and western blot analysis (with two different parkin antibodies) as described in the Materials and Methods section. The analysis demonstrated a complete loss of parkin protein in PKO rats (Fig. 1A).

### The effect of parkin deficit on body weight and core body temperature

In contrast to *Park2*^−/−^ knockout mice^11,19,20^, the *Park2*^−/−^ knockout rats displayed normal core body temperature (Fig. 1B) and higher body weight between the ages of 1 and 2 months than the age-matched WT controls (+12.6%, Student’s t-test, p < 0.001, n = 12) (Fig. 1C).

### The effect of parkin deficit on cytoskeletal protein levels

Both α-tubulin and ß-tubulin are substrates of parkin^21^. Actins were not demonstrated to be substrates of parkin^22^ but parkin is known to interact with actin filaments^23^. To assess whether the loss of parkin protein affects α-tubulin and ß-actin protein levels, whole synaptosomes and synaptosomal fractions were prepared from the striata of 2 month-old PKO and WT rats and quantitatively analyzed in relation to total protein levels (estimated by Ponceau S staining). We found similar levels of α-tubulin as well as ß-actin in lysates as well as in whole synaptosomes from the PKO and WT rats. In contrast, ß-actin immunoreactivity in synaptosomal fractions differed between the two groups. In the PKO group, ß-actin immunoreactivity was higher in the membrane-endosomal fraction (+20%, p < 0.05, Student’s t-test, n = 4) and lower in the cytosol-vesicular fraction (−16%, p > 0.1, Student’s t-test, n = 4). Similar trends were detected for immunoreactivity of α-tubulin; however, the changes did not reached statistical significance (not shown). Since neither ß-actin nor α-tubulin proved to be a reliable gel loading control in the experiments employing striatal synaptosomal fractions; total protein content (Ponceau S staining) was used as a loading control in experiments involving the fractions. ß-Actin was used as a loading control when total synaptosomal fractions or whole tissue lysates were assessed.

### The effect of parkin deficit on synthesis, storage, and metabolism of striatal dopamine

**Figure 2:**
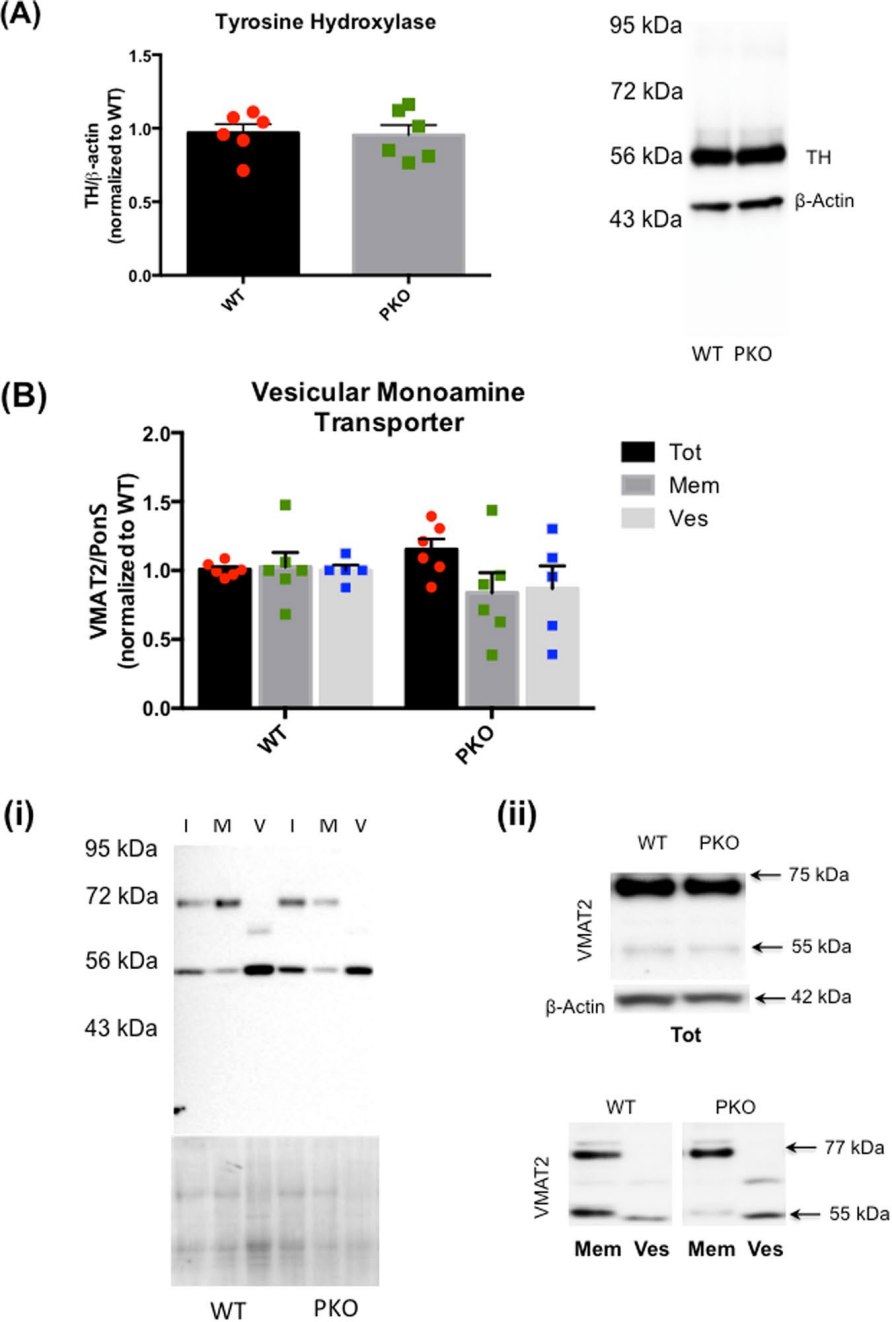
The effect of parkin protein loss on synthesis and storage of striatal dopamine. Whole synaptosomes and synaptosomal fractions were isolated from striata of the 2 month-old parkin knockout (PKO) and wild type (WT) Long Evans male rats. Compared with the WT controls, there was no significant difference in the immunoreactivity of (**A**) TH (detected at ~60 kDa) or (**B**) VMAT2 (detected at ~70–75 kDa and 55 kDa, representing glycosylated and partially glycosylated VMAT2, respectively) in synaptosomal fractions from the striata of WT and PKO rats. An additional band of ~65 kDa was apparent in the vesicular fraction of VMAt2. (**2Bi**) A representative blot containing all synaptosomal fractions and the corresponding total protein content. (**2Bii**) VMAT2 levels in total synaptosomal fraction and corresponding ß-actin levels as well as another representative blot showing VMAT2 in synaptosomal fractions. Abbreviations: Mem, membrane fraction; Ves, vesicular fraction; VMAT2, vesicular monoamine transporter 2; TH, tyrosine hydroxylase; Tot, total synaptosomal fraction. The data are expressed as mean ± SEM.

Whole synaptosomes and synaptosomal fractions were prepared from the striata of 2 month-old PKO and WT rats and assessed for the levels of TH, a rate-limiting enzyme in DA synthesis and for the levels of VMAT2, a vesicular transporter packaging DA to the storage vesicles. The striatal content of DA and its metabolites, 3,4-dihydroxyphenylacetic acid (DOPAC), homovanillic acid (HVA), and (3-methoxytyramine (3-MT) was assessed in striatal lysates. We found no significant difference in the immunoreactivity of TH, detected at ~60 kDa, between the PKO rats and their WT counterparts (Fig. 2A). The immunoreactivity of VMAT2 was detected at ~70–75 kDa and 55 kDa, representing glycosylated and partially glycosylated form of VMAT2, respectively. This pattern of VMAT2 reactivity has been reported in the rat^24,25^ and human^26,27^ brain. The loss of parkin protein did not affect total VMAT2 levels (calculated as sum of all bands) or subcellular distribution of VMAT2 (Fig. 2B). Figure 2B(i) shows a representative blot containing all fractions together on the same blot and the corresponding total protein content. Figure 2B(ii) shows total levels of VMAT2 and corresponding ß-actin levels as well as another representative blot showing VMAT2 levels in synaptosomal fractions. Of note, a ~65 kDa band was observed in the vesicular fraction instead of the 75–80 kDa band, probably representing another glycosylated species of the VMAT2. No statistically significant differences were found in the levels of DA and its metabolites in the PKO rats compared to the WT controls (Table 1). To assess metabolism of intracellular DA, the DOPAC/DA ratios were calculated and found not to differ significantly between the genotypes (Table 1). To assess metabolism of extracellular DA, the 3-MT/DA and 3-MT/DOPAC ratios were calculated. Both ratios were similar in the PKO group and the WT group (Table 1), suggesting unaltered DA release and metabolism outside DAergic terminals. Overall, the data suggested normal DA synthesis, storage, and metabolism in the striatum of 2 month-old PKO rats.

**Table 1:** Dopamine (DA), DA metabolites and DA metabolism in wild type (WT) and parkin knockout (PKO) 2 month-old rats.

	WT	PKO
DA	111 ± 6	109 ± 14
DOPAC	13.4 ± 1.0	15.1 ± 1.7
HVA	2.68 ± 0.19	2.69 ± 0.10
3-MT	0.993 ± 0.090	0.935 ± 0.209
DOPAC/DA	0.104 ± 0.007	0.105 ± 0.009
3-MT/DA	0.009 ± 0.012	0.012 ± 0.018
3-MT/DOPAC	0.079 ± 0.02	0.079 ± 0.017

Values are expressed as mean ± SEM in pg/µg protein.

### Loss of parkin protein decreases the activity of monoamine oxidase B in the rat striatum

**Figure 3:**
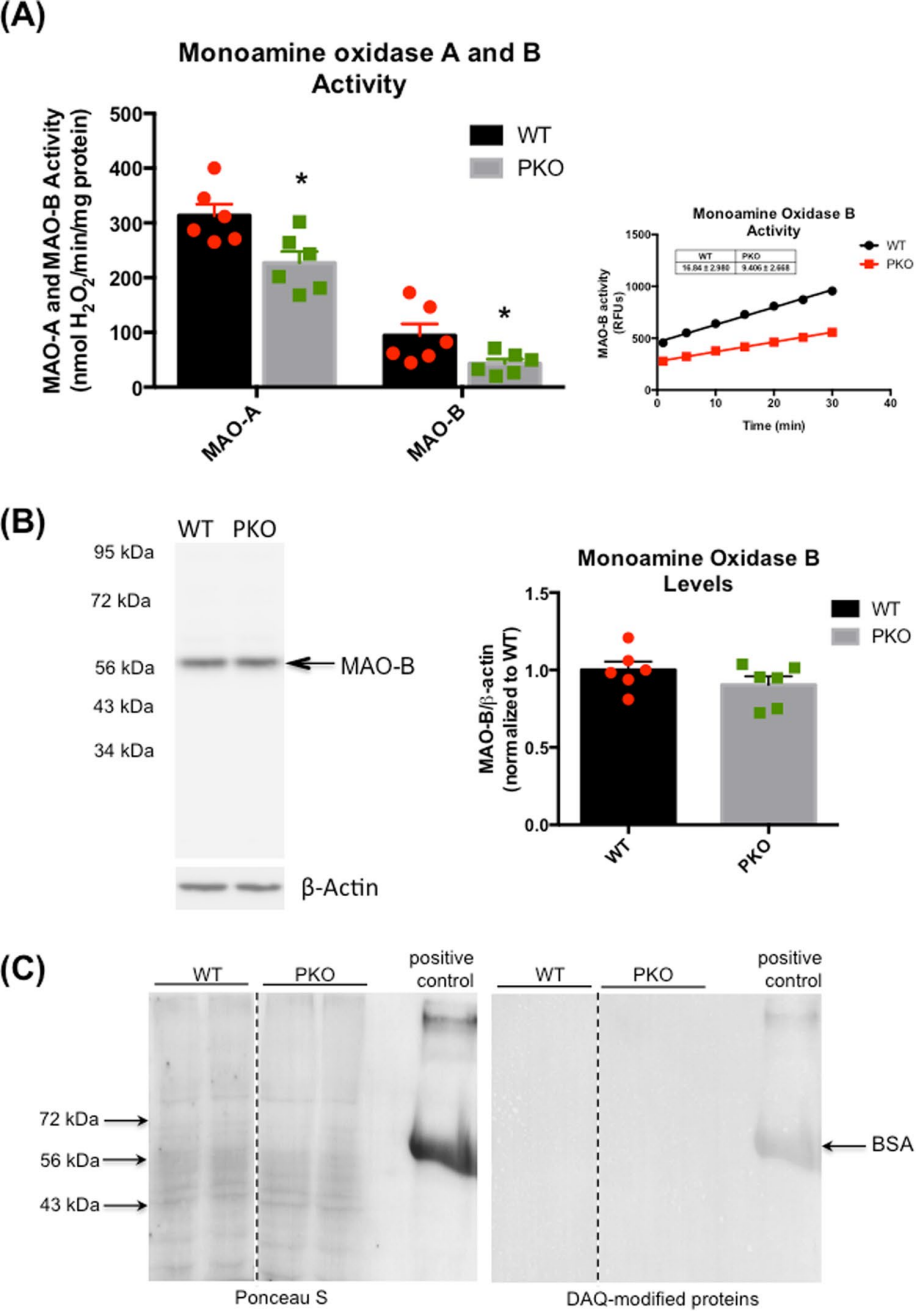
The effect of parkin protein loss on striatal dopamine metabolism. (**A**,**B**) Mitochondria isolated from the striata of parkin knockout (PKO) and wild type (WT) rats, monitoring the rate of H_2_O_2_ production over 30 min (Fig. 3A, graph on the right). (**A**) In the PKO rats, striatal activity of MAO-A and MAO-B was significantly reduced (−27% and −46%, respectively, p < 0.05, Student’s t-test, n = 6) compared to the WT controls. MAO-B activity, measured with p-tyramine as a substrate for both isoforms, was linear over measured 30 min time period. (**B**) SDS-PAGE and western blotting analysis revealed no difference in the levels of MAO-B protein (n = 6) . (**C**) Lack of immunoreactivity for proteins modified by DA quinones in the striatal lysates of WT and PKO rats; DA quinone-modified bovine serum albumin serves as a positive control. The data are expresses as mean ± SEM. *p < 0.05. Abbreviations: BSA, bovine serum albumin; DA, dopamine; DAQ, dopamine quinone; MAO, monoamine oxidase.

To further characterize the effect of *Park2* knockout on DA metabolism, we measured activities of MAO-A and MAO-B in mitochondria isolated from the striata of PKO and WT rats, monitoring the rate of H_2_O_2_ production over 30 min (Fig. 3A, graph on the right). In rat brain, MAO-A is a major isoform metabolizing DA with MAO-B being minimally involved in DA metabolism, preferentially metabolizing ß-PEA^28–32^. Striatal activities of both monoamine oxidases, particularly of MAO-B, were significantly reduced (−27% and −46%, respectively, p < 0.05, Student’s t-test, n = 6) in the PKO rats compared to the WT controls (Fig. 3A, graph on the left). This result was unexpected as available literature data reports increased levels, or activities, of MAO-A and MAO-B upon parkin gene knockout^33,34^. The assessment of MAO-B protein levels in the striatum (by calculation of immunoreactivity of the ~58 kDa band) revealed no difference in MAO-B protein levels between the knockouts and the controls (Fig. 3B) .

The decreased activity of MAO-A introduced a possibility of increased levels of cytoplasmic DA and DA-mediated oxidative stress in the striatum of PKO rats. To investigate this possibility, the levels of DA quinones and 4-HNE, a by-product of lipid peroxidation, were assessed in the striata. There was no detectable immunoreactivity for DA quinone-modified proteins in striatal synaptosomes or lysates from the WT or PKO rats (Fig. 3C). The immunoreactivity for 4-HNE-conjugated proteins in striatal synaptosomes was also no different in the PKO rats than in the WT controls (not shown).

### Loss of parkin protein alters the levels of ß-phenylethylamine and trace amine-associated receptor 1 in the rat striatum

**Figure 4:**
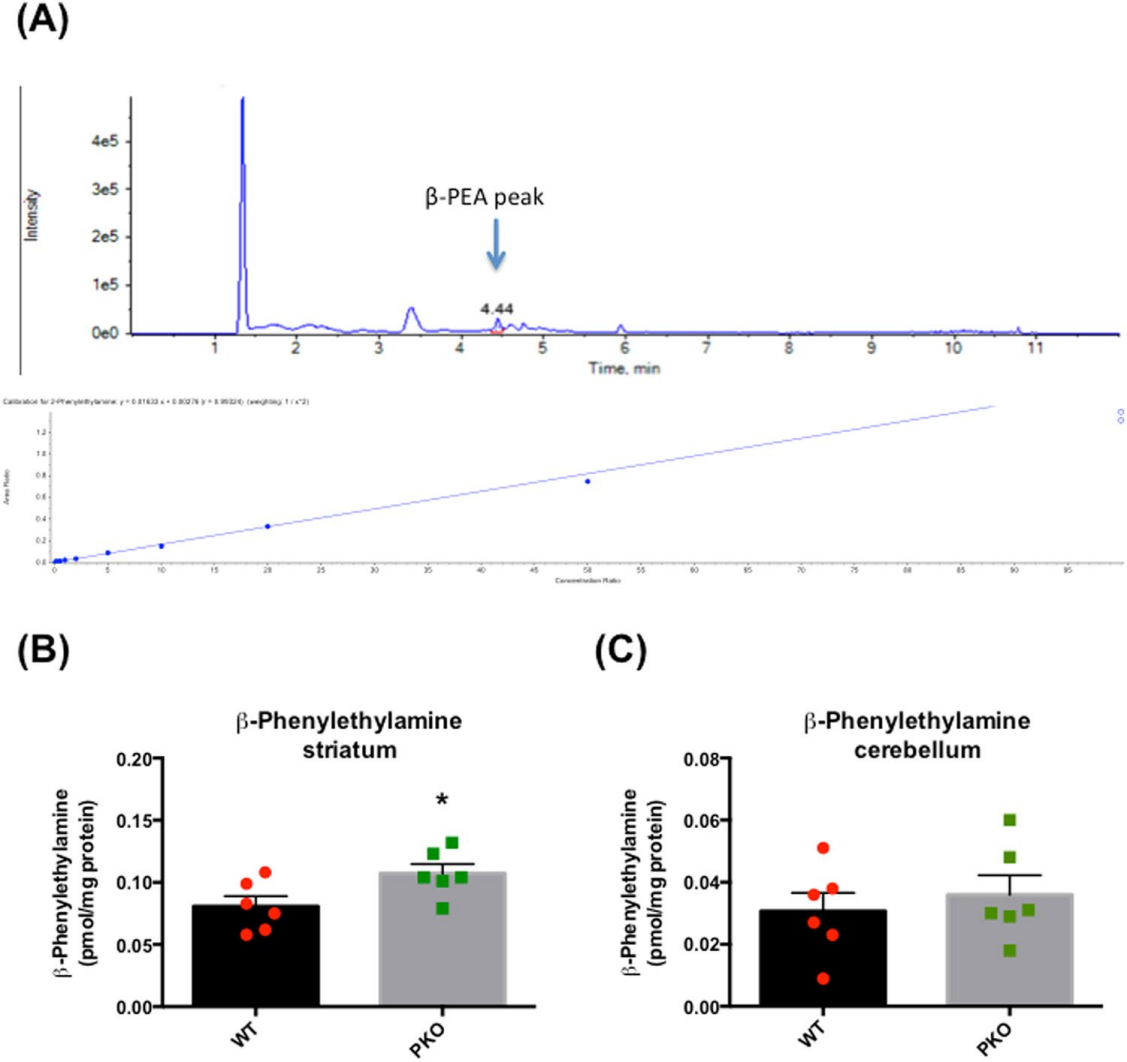
The effect of parkin protein loss on the striatal levels of ß-phenylethylamine. Tissue lysates were prepared from striata of the 2 month-old parkin knockout (PKO) and wild type (WT) Long Evans male rats. (**A**) A representative peak of ß-phenylethylamine (ß-PEA) and the standard curve. (**B,C**) The levels of ß-PEA were significantly higher in the striatum (+33%, p < 0.05, n = 6) (**B**) but not in the cerebellum (p > 0.1, n = 6) (**C**) in the PKO rats as compared to the WT rats. The data are expressed as mean ± SEM. *p < 0.05, two-way ANOVA with Holm-Sidak’s *post hoc* test.

While ß-PEA is a preferred substrate for MAO-B in rat brain^31,32^, 5-HT is a preferred substrate for MAO-A^29^. The levels of 5-HT, its metabolite 5-hydroxyindoleacetic acid (5-HIAA), and 5-HIAA/5-HT ratio did not significantly differ from the control values (5-HT: 6.16 ± 0.47 *vs*. 5.19 ± 1.27 pg/µg protein; 5-HIAA: 4.60 ± 0.32 *vs*. 4.16 ± 0.58 pg/µg protein; 5-HIAA/5-HT: 0.757 ± 0.028 *vs*. 0.963 ± 0.170, in PKO *vs*. WT rats). The ß-PEA data was highly variable, most likely due to the fact that ß-PEA levels are low in the brain and, therefore, difficult to assess with precision (Fig. 4A). The levels of ß-PEA were significantly higher in striata from the PKO rats compared to the WT rats (+33%, PKO: 0.107 ± 0.008 *vs*. WT: 0.081 ± 0.008 pmol/mg protein, p < 0.05, n = 6) (Fig. 4B). ß-PEA levels in the DA-poor cerebellum (assessed to determine whether the changes in ß-PEA are specific to the DA-rich striatum) were not significantly different between the genotypes (PKO: 0.036 ± 0.006 *vs*. WT: 0.031 ± 0.006 pmol/mg protein, n = 6) (Fig. 4C). The ß-PEA data was analyzed by two-way ANOVA followed by Holm-Sidak’s *post hoc* test. There was a significant effect of genotype (*F*(1, 20) = 5.11, p < 0.05) and a significant effect of brain area (*F*(1, 20) = 74.9, p < 0.0001).

**Figure 5:**
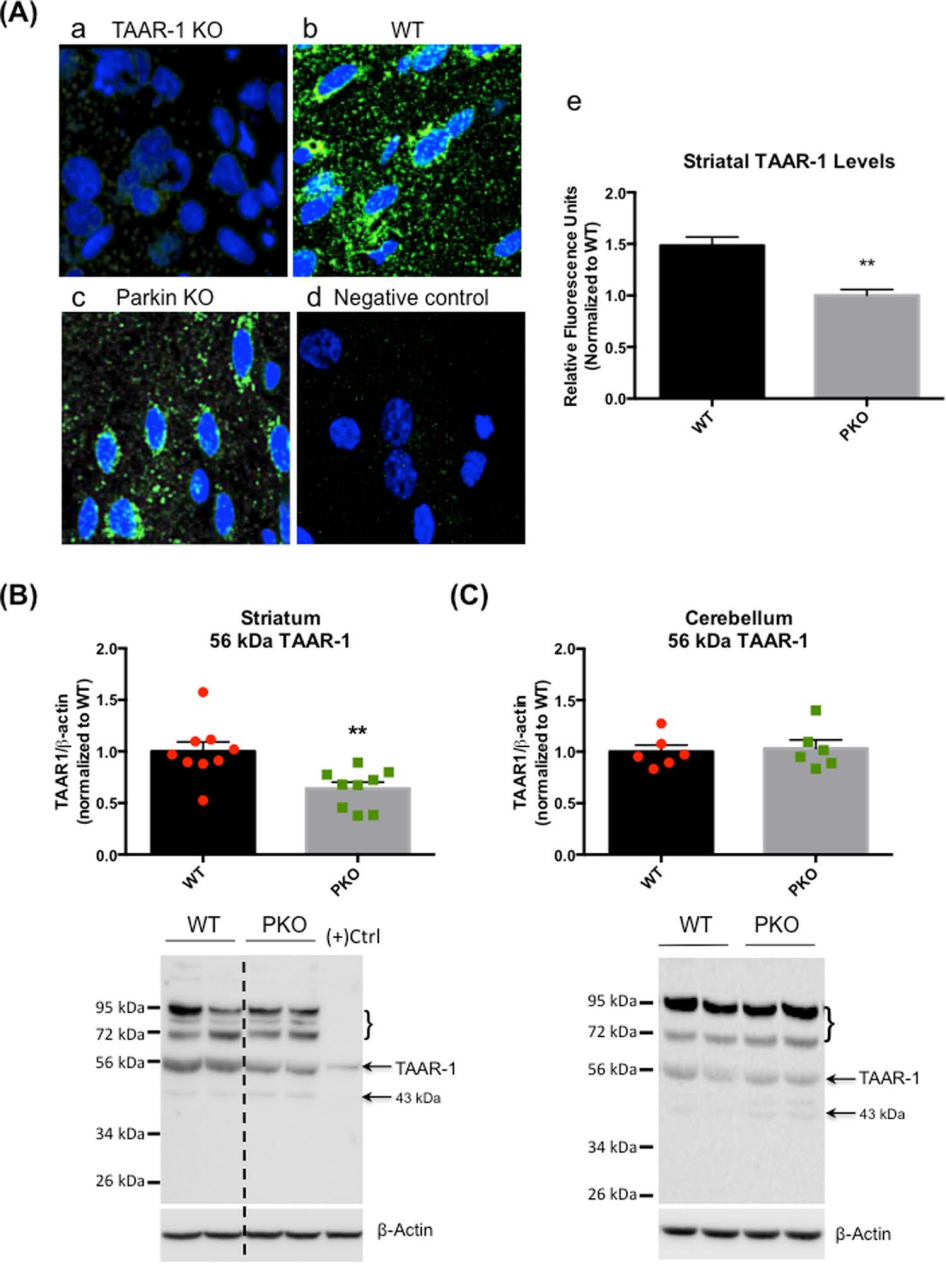
The effect of parkin protein loss on the striatal levels of trace amine-associated receptor 1. (**A**) Tissue lysates were prepared from striata of the 2 month-old parkin knockout (PKO) and wild type (WT) Long Evans male rats. (a) Validation of antibody against trace amine-associated receptor 1 (TAAR-1). There was no signal for TAAR-1 receptor in striatal slices from *TAAR1*^−/−^ knockout rats. (b, c) Immunochemical staining of striatal slices from the WT rats and the PKO rats with the TAAR-1 antibody revealed diffuse cytoplasmic signal. (d) The no-primary antibody negative control for TAAR-1 antibody. (e) Quantification of TAAR-1 immunostaining revealed decreased levels of the receptor in the PKO rats compared to the WT controls (−33%, Student’s t-test, p < 0.01, n = 3). (**B**) Assessment of TAAR-1 levels in striatal lysates by SDS-PAGE and western blotting. The immunoreactivity of the 56 kDa band (glycosylated TAAR-1) was significantly decreased (−36%, p < 0.01, two-way ANOVA followed by Holm-Sidak’s *post hoc* test, n = 9) in the striatum of PKO rats compared to the WT controls. (**C**) The immunoreactivity of 56 kDa TAAR-1 band in the cerebellum of PKO rats did not show a decrease. The data are expresses as mean ± SEM, **p < 0.001.

The TAAR-1 receptor is a member of recently identified TAAR family of G protein-coupled receptors^35^ and a receptor for trace amines such as ß-PEA as well as a receptor for amphetamines^36^. TAAR-1 localizes intracellularly in presynaptic and postsynaptic neuronal elements^37^. Disease states associated with impaired TAAR-1 function include drug abuse, obesity, diabetes, schizophrenia, and PD^38–40^. We next assessed whether the increase in ß-PEA levels affected protein levels of TAAR-1 in striatal and cerebellar lysates from the PKO rats. First, we validated the anti-TAAR-1 antibody. As shown in Fig. 5Aa, no signal for TAAR-1 receptor was observed in striatal slices from *TAAR1*^−/−^ knockout rats. Immunochemical staining of striatal slices from the WT and PKO rats with the TAAR-1 antibody revealed diffuse cytoplasmic signal (Fig. 5Ac), which was decreased in the knockouts compared to the controls (−33%, Student’s t-test, p < 0.01, n = 3) (Fig. 5Ae). Subsequently, the same antibody was used in SDS-PAGE/western blotting to confirm the decrease in TAAR-1 receptor levels. The expected molecular weight for the TAAR-1 is ~39 kDa. This bare form of the receptor is usually observed at 34–39 kDa and its glycosylated form is observed at 43–52 kDa. In our hands, the TAAR-1 immunoreactivity was detected at ~43 kDa and ~56 kDa band, with the latter most likely reflecting glycosylated form of the receptor^41^. To determine whether parkin deficit differentially affects TAAR-1 in different brain areas, the levels of TAAR-1 were assessed in DA-rich striatum and DA-poor cerebellum in the PKO and the WT rats. Two-way ANOVA revealed a significant effect of brain area (*F*(1, 26) = 5.77, p < 0.05) but not of genotype (*F*(1, 26) = 4.17, p = 0.051). However, there was a significant area x genotype interaction (*F*(1, 26) = 5.77, p < 0.05), indicating a difference between genotypes in the striatum but not in the cerebellum. The immunoreactivity of the 56-kDa band was significantly decreased (−36%, p < 0.01, two-way ANOVA followed by Holm-Sidak’s *post hoc* test, n = 9) in the striatum of the PKO rats compared to the WT rats (Fig. 5B). The immunoreactivity of 56 kDa TAAR-1 band in the cerebellum of the PKO rats did not show a decrease (Fig. 5C). In the PKO rats, the immunoreactivity of unglycosylated TAAR-1 was no different from the control values (Fig. 5B,C). In addition to 43 kDa and 56 kDa bands, the anti-TAAR-1 antibody detected bands above 72 kDa in both PKO and WT samples. These bands are most likely non-specific bands.

### Loss of parkin protein decreases the levels of postsynaptic dopamine D2 receptor in the rat striatum

**Figure 6:**
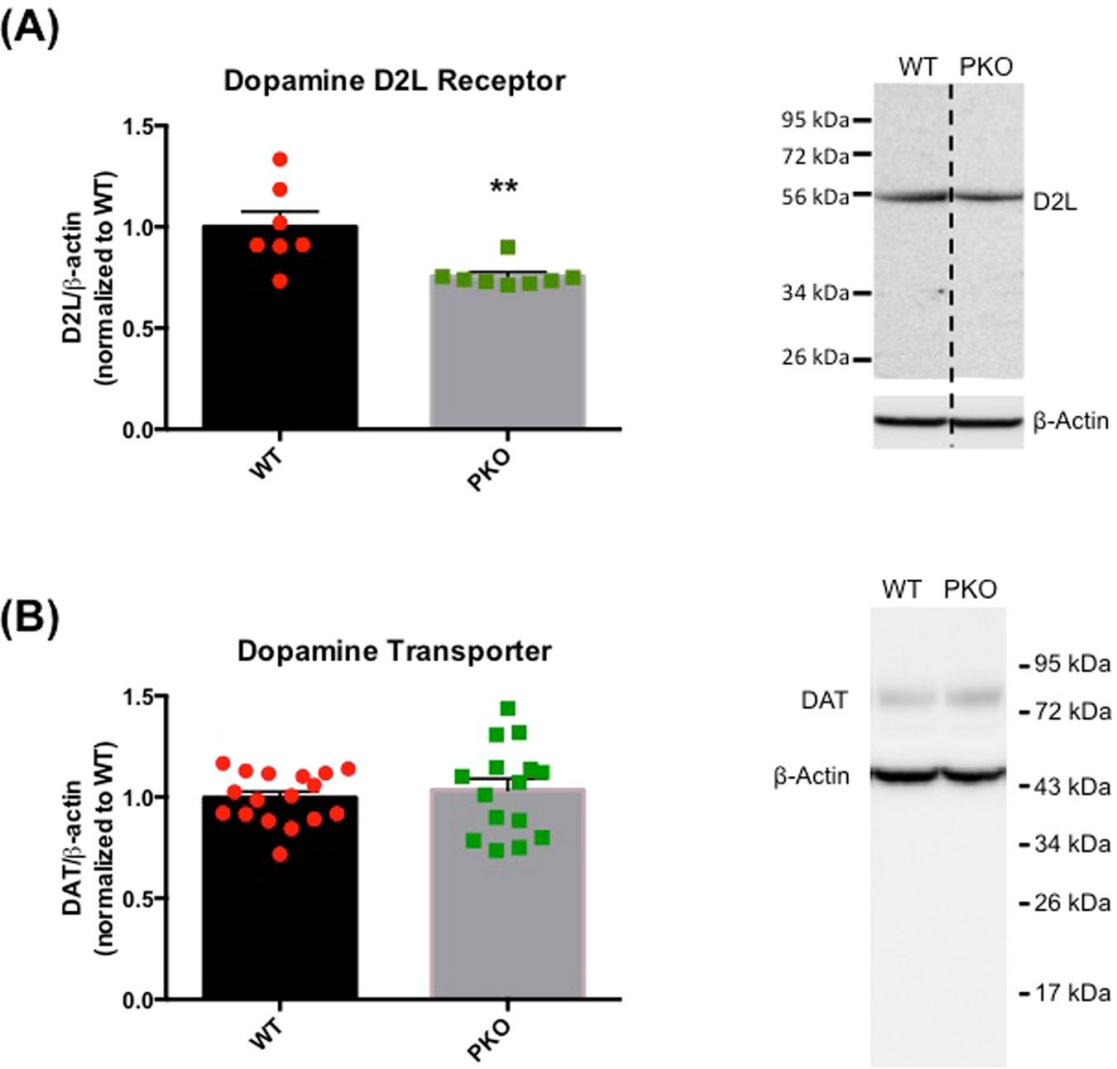
The effect of parkin protein loss on the levels of postsynaptic dopamine D2 receptor and dopamine transporter in the rat striatum. Tissue lysates were prepared form striata of the 2 month-old parkin knockout (PKO) and wild type (WT) Long Evans male rats. (**A**) The levels of postsynaptic D2 receptor (D2L) (~55 kDa) were significantly decreased (−27%, p < 0.01, Student’s t-test, n = 7–9) in the striatum of PKO rats compared to the striatum of WT controls. (**B**) The immunoreactivity of glycosylated dopamine transporter (DAT) (detected at ~70 kDa) did not significantly differ between the PKO and WT phenotypes in the majority of the PKO rats (n = 15–17). The data are expressed as mean ± SEM, **p < 0.01.

The TAAR-1 receptor is known to modulate presynaptic and postsynaptic DA D2 receptors while ß-PEA was reported to modulate postsynaptic D2 receptor (D2L)^42–44^. Therefore, we next evaluated the total levels of DA D2 receptor (D2(S + L)), and the levels of its postsynaptic isoform, i.e. D2L receptor, in the striatal lysates from the WT and PKO rats. As previously reported by us^25^, the antibody against D2L receptor detected partially glycosylated D2L receptor (~55 kDa) whereas the antibody against presynaptic and postsynaptic D2 receptor (D2(S + L)) recognized D2 receptor at different states of glycosylation (50, 72 and ~80–100 kDa). Both antibodies have previously been used in other studies (e.g.^45–47^). Compared to the WT controls, the levels of D2L receptor were significantly decreased in striatal lysates from the PKO rats (−27%, p < 0.01, Student’s t-test, n = 7) (Fig. 6A) whereas the total levels of D2 receptor (S + L) where not significantly changed (not shown). These results suggest that the levels of presynaptic D2 receptor (D2S) may be increased in the striatum of the PKO rats.

The DAT is a plasma membrane transporter responsible for uptake of DA back into DAergic terminals. The levels of DAT were assessed to estimate DA uptake. The antibody against the DAT detected fully glycosylated form of the transporter (~70 kDa)^25^. The immunoreactivity of striatal DAT was not significantly different than the control levels of the transporter in most of the PKO rats (Fig. 6B).

### The effects of parkin deficit on the expression of *TAAR1*, *DRD2*, and *MAOB* gene

**Figure 7:**
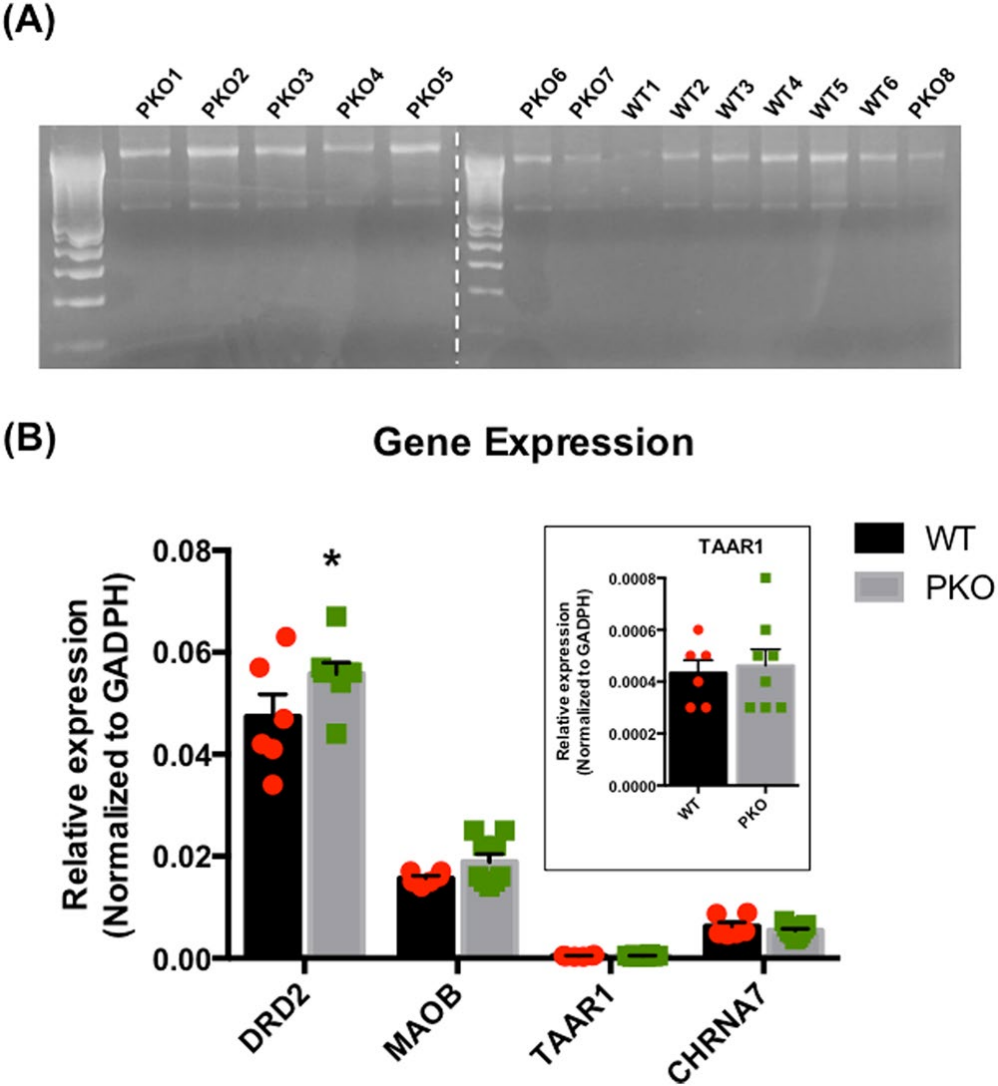
The effects of parkin deficit on the expression of *TAAR1*, *DRD2*, or *MAOB* gene. (**A**) The mRNA was isolated from the striata of 2 month-old parkin knockout (PKO) and wild type (WT) rats and assessed for quality. (**B**) Real-time Polymerase Chain Reaction (qPCR) analysis showed that the levels of *TAAR1* and *MAOB* mRNA did not significantly differ form the control values whereas the levels of *DRD2* mRNA were significantly increased (+18%, p < 0.05, two-way ANOVA followed by Holm-Sidak’s *post hoc* test, n = 6–9). The levels of “control” *CHRNA7* gene were also at the control values. The data are expressed as mean ± SEM, *p < 0.05. Abbreviations: CHRNA7, acetylcholine receptor subunit; D2R2, dopamine D2 receptor; alpha-7; MAOB, monoamine oxidase B; TAAR1, trace amine-associated receptor 1.

To determine whether the decreases in TAAR-1 and D2L levels were due to decreases in their gene expression, we employed the qPCR. The data was analyzed by delta CT method and normalized to WT controls. Two-way ANOVA revealed a significant effect of genotype (*F*(1, 50) = 4.88, p < 0.05) as well as gene (*F*(3, 50) = 343, p < 0.0001) and significant genotype x gene interaction (*F*(3, 50) = 2.87, p < 0.05). In contrast to our expectations, the levels of *TAAR1* mRNA did not significantly differ from the control values whereas the levels of *DRD2* mRNA were significantly increased (+18%, p < 0.05, two-way ANOVA followed by Holm-Sidak’s *post hoc* test, n = 6–9) (Fig. 7). The acetylcholine receptor subunit alpha-7 (*CHRNA7*) gene expression served as a non-DAergic gene control.

### Loss of parkin protein decreases protein-protein interactions of TDP-43 protein

**Figure 8:**
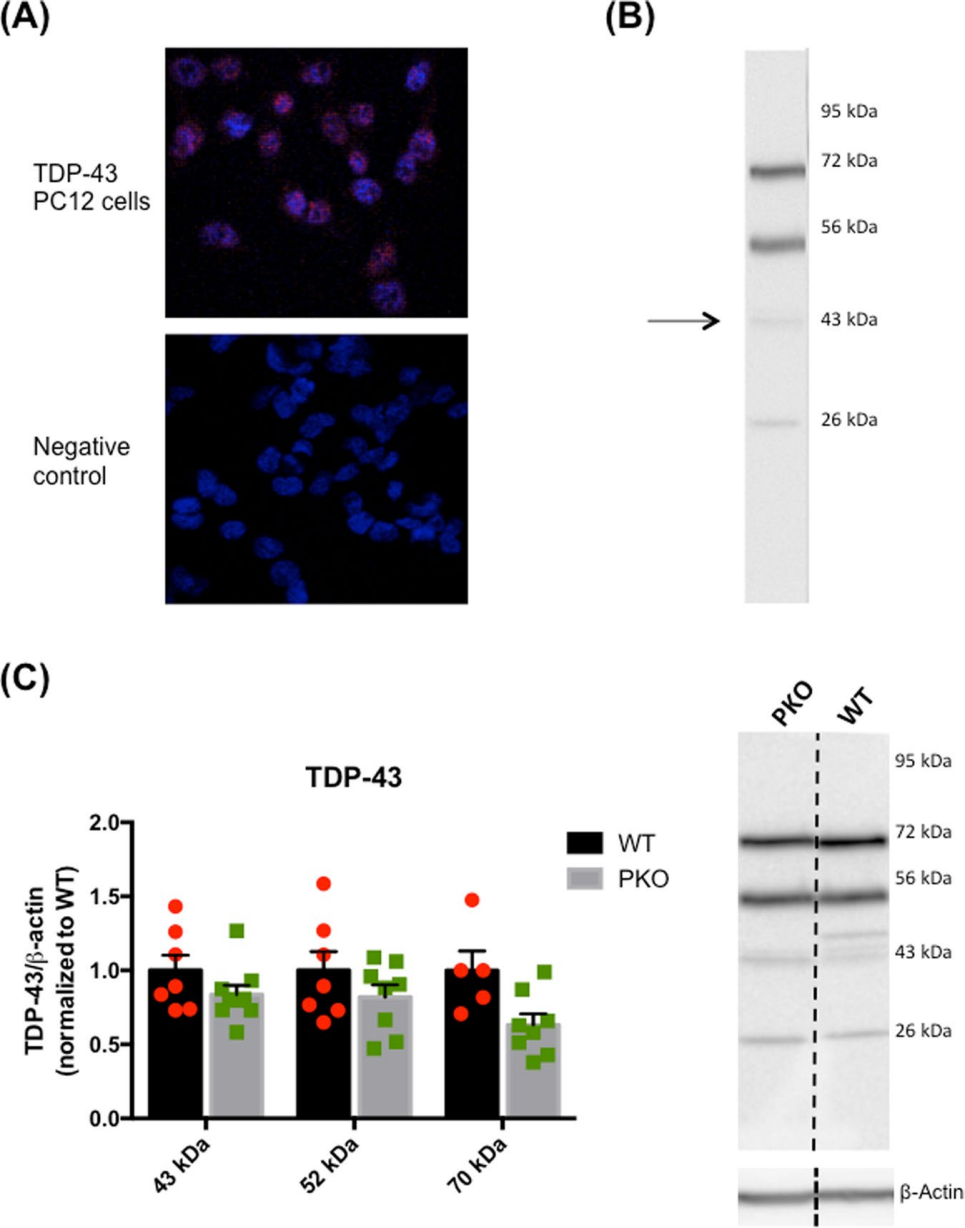
The effect of parkin protein loss on the levels of transactivation DNA-binding protein (TDP-43). (**A**) The anti-TDP-43 antibody was validated in PC12 cells and found, as expected, in the nucleus. (**B**) The immunoreactivity of TDP-43 was detected as 43 kDa band and two other bands, ~52 kDa and ~70 kDa band. A weak, potentially non-specific band of ~26 KDa was also observed on the membranes. (**C**) The 43 kDa and 52 kDa bands did not differ in optical density between the PKO and WT rats while 70 kDa band showed a trend for lower density in the PKO rats compared to the controls (−37%, p < 0.1, two-way ANOVA with Holm-Sidak’s *post hoc* test, n = 6–9). The data are expressed as mean ± SEM).

Altered levels of TAAR-1, D2L, or MAO-B without similar alterations in their gene expression suggested that parkin deficiency might affect the process of translation. To determine whether the loss of parkin affects mRNA-protein translation in the striatum of PKO rats, we assessed the levels of transactivation DNA-binding protein (TDP-43), a DNA/RNA-binding protein involved in regulation of transcription and translation processes^48,49^. TDP-43 is ubiquitinated by parkin^50^ and its levels are increased in parkin knockout mice^51^. The anti-TDP-43 antibody was validated in PC12 cells and TDP-43 was found, as expected, in the nucleus (Fig. 8A). We detected two main bands, ~52 kDa and ~70 kDa band (Fig. 8B). Neither of these bands represented TDP-43-RNA or TDP-43-DNA complex as treatment of the samples with benzonase nuclease did not result in a single 43 kDa band (not shown). Similar bands were observed in other studies employing WT samples^50,52,53^. The 52 kDa band is likely ubiquitinated TDP-43^53^ while the 70 kDa band may be a complex of TDP-43 with one of its interacting protein partners. In addition to the weak ~43 kDa band, a weak, likely non-specific, band of ~26 kDa was also observed on the membranes (Fig. 8B). Two-way ANOVA revealed a main effect of genotype (*F*(1, 38) = 9.24, p < 0.01) (Fig. 8C). The 43 kDa and 52 kDa bands did not differ in optical density between the PKO and WT rats while 70 kDa band was of lower density in the PKO rats compared to the controls (−37%, p < 0.01, two-way ANOVA with Holm-Sidak’s *post hoc* test, n = 6–9) (Fig. 8C). The total intensity of the three bands was slightly decreased in the striatum of PKO rats compared to WT controls (WT: 1.00 ± 0.10 *vs.* PKO: 0.76 ± 0.06; −24%, p < 0.05, Student’s t-test).

### Loss of parkin protein decreases locomotor response to methamphetamine challenge

**Figure 9:**
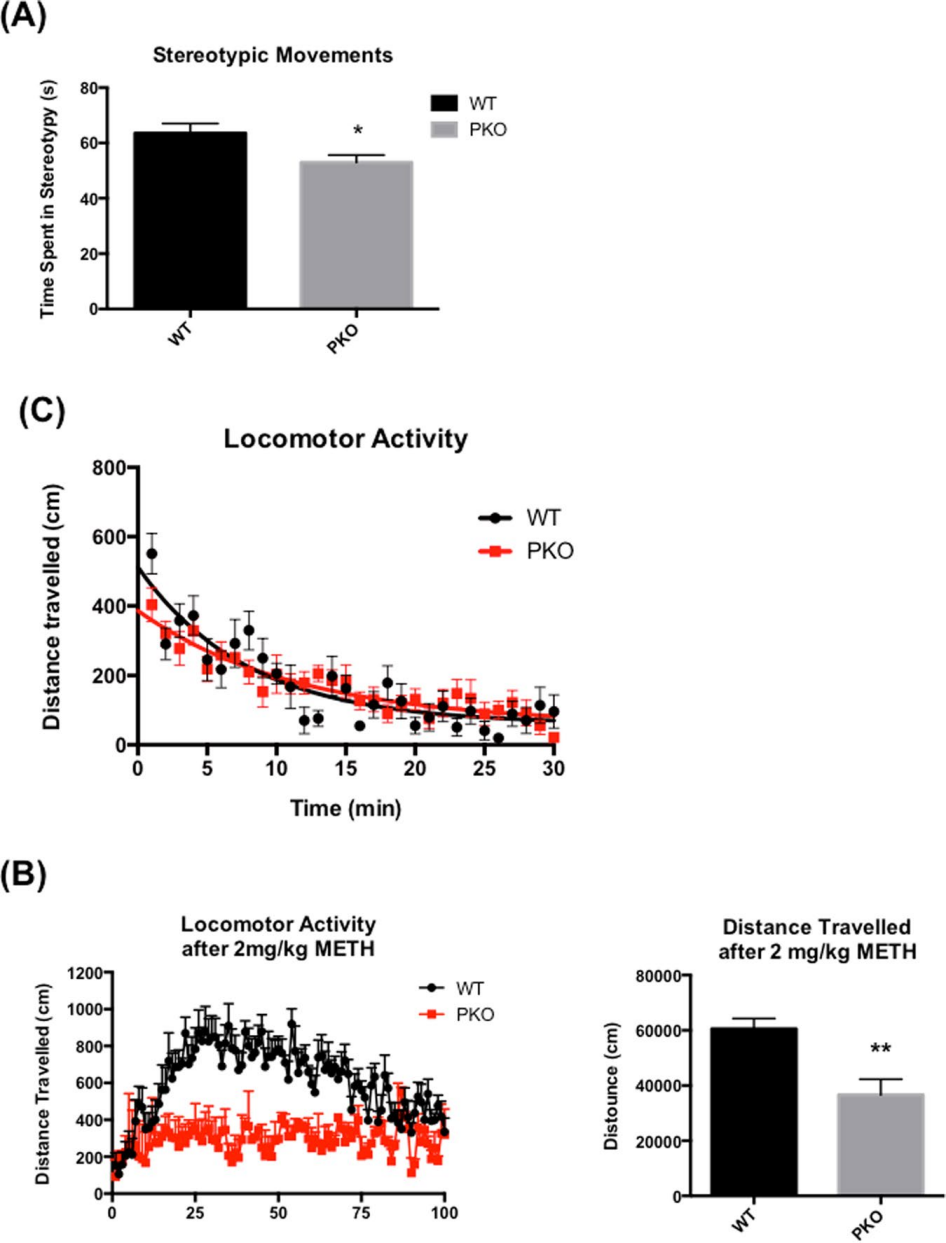
The effect of parkin protein loss on motor behaviors. The parkin knockout (PKO) and wild type (WT) Long Evans male rats were assessed for locomotor activity, stereotypic movements at the response to 2 mg/kg methamphetamine at the age of 2 months. (**A**) Compared to the WT controls, the PKO rats showed a significantly decreased stereotypic movements (−18%, p < 0.05, Student’s t-test, n = 6). (**B**) There was no difference between PKO and WT rats in distance traveled over 30 min. (**C**) The locomotor response of PKO to 2 mg/kg dose of methamphetamine (METH) was significantly lower than the response of WT controls (−39%, Student’s t-test, p < 0.01, n = 6). The data are expressed as mean ± SEM. *p < 0.05, **p < 0.01.

Next, we tested whether the observed alterations in DAergic and trace aminergic system affected motor activity in PKO rats. Small stereotypic movements (including grooming) measured within 30 min time frame were significantly decreased in the PKO rats compared to the controls (−18%, Student’s t-test p < 0.05, n = 6) (Fig. 9A). There was no difference between the PKO and WT rats in distance traveled over 30 min timeframe (Fig. 9B). However, the locomotor response of PKO to 2 mg/kg injection methamphetamine was significantly lower than the response of WT controls (−39%, Student’s t-test, n = 6) (Fig. 9C).

## Discussion

Parkin is a neuroprotective ubiquitin protein ligase with multiple substrates and functions, which contributes to development of PD^54,55^. Surprisingly, neither PKO mice (between 3 and 24 months of age)^8^ nor PKO rats (between 4 and 8 months of age) display nigrostriatal system degeneration^9^. The present study aimed to characterize DAergic system in the striatum of 2 month-old PKO Long Evans male rats and demonstrated that homogenous knockout of *Park2* gene (exon 4) and loss of parkin protein were associated with an increase in striatal levels of ß-PEA and deficits in MAO-B/A, D2L receptor and TAAR-1 receptor in the striatum. The changes were not associated with deficits in presynaptic DAergic markers nor were they associated with significant motor impairments. They were associated with a decrease in methamphetamine-evoked DA release.

Three exon 3-deleted PKO mouse lines (20 days to 12 months of age) displayed significantly reduced body weight^11,19,20,56^ that was concomitant with decreased body temperature in one of these studies^19^. In addition, the average body weight of PKO mice with a mixed C57BL6/129S4 background described by Perez and Palmiter was decreased compared to controls at 6 months of age but not younger or older^57^. However, this was not the case for mice with a 129S4 genetic background generated by the same group. The 2 month-old exon 4-deleted male Long Evans rats used in our study displayed unaltered core body temperature and increased body weight, supporting the existing evidence for age, genetic background, and exon-mediated differences in PKO rodents. Importantly, our findings add to this knowledge the existence of species differences. Exon 3-deleted 3 month-old C57BL6/129S4 PKO mice fed food rich in fat and cholesterol resisted weight gain via altered fat uptake and lipid metabolism^56^. It is possible that the young adult PKO rats have different fat uptake and/or lipid metabolism than the young adult PKO mice or that these processes do not largely depend on parkin function in rats as they do in mice. Alternatively, high body weight in PKO rats is related to decreased levels of TDP-43 protein in the brain and liver. TDP-43 regulates the expression and protein levels of parkin^49,50,58^. Conversely, parkin is known to regulate TDP-43 function^50,51^. TDP-43 mutant knock-in mice display a loss of body weight compared to WT controls^59^, suggesting that TDP-43 knockout or knockdown would result in a weight gain. Along different lines, TAAR1 activation has been shown to prevent olanzapine-induced weight gain^38^; therefore, a decrease in TAAR-1 protein levels in PKO rats may contribute to higher than normal body weight in these rats.

In agreement with previous studies that did not find significant changes in the levels of α-tubulin, a substrate of parkin, in striata of PKO mice^60,61^, we found no changes in α-tubulin levels in striatal synaptosomes from the PKO rats compared to the WT controls. Parkin was reported to interact with actin filaments^23^; therefore, the increased levels of ß-actin in the endosomal-membrane fraction may reflect a compensatory increase in ß-actin interacting with the plasma membrane.

Neurochemical quantification of striatal tissue from young adult (3 month-old) PKO mice reported normal levels of DA and contradictory results on the levels of DOPAC and HVA in the striatum. Thus, increased DOPAC and/or HVA levels were reported in two studies^62,63^ with no change reported in one study^11^. Our finding of unchanged levels of DA, DOPAC and HVA agrees with the latter report in mice and with a study on 4–8 month-old PKO rats that also found no alterations in the same DAergic markers^9^. The ratio DOPAC/DA and 3-MT/DA are considered to reflect intracellular *vs*. extracellular DA metabolism (or stored and released DA), respectively. Our finding of unaltered content of striatal DA and unaltered DA intracellular metabolism (manifested by unaltered DOPAC/DA ratio) suggest normal DA synthesis, storage and uptake in the absence of parkin protein. In agreement with this conclusion, we found no difference in the levels of TH, a rate-limiting enzyme in DA synthesis, in the levels of DAT, a transporter responsible for DA uptake back to the terminal, or in the levels of VMAT2, an antiporter packaging DA to the storage vesicles, between the PKO and the WT rats. Our TH results agree with those obtained by other laboratories^9,11,19,63^. Regarding the VMAT2, one of the previous studies found a decrease while another study found no change in striatal VMAT2 levels in old mice (11 month-old and 12 month-old mice, respectively)^19,64^. Of note, we did not measure the rate of DA uptake by the VMAT2 neither did we measure DA content in DA storage vesicles. Therefore, as suggested by a study in PKO mice^19^, it is possible that PKO rats have different sizes of stored and cytosolic DA pools than their WT counterparts.

Lack of an increase in DA levels in view of decreased MAO-A activity could be explained by the fact that MAO-A activity was measured under the *V*_max_ conditions and, therefore, reflects the levels rather than the function of the enzyme. The concentration of DA in the cytoplasm is below the *K*_m_ value, i.e. affinity of MAO-A for DA. Consequently, intracellular DA metabolism could have increased regardless of the decrease in the levels of MAO-A. Alternatively or in addition, DA synthesis could have decreased due to decreased TH affinity for DA, without changes in TH levels. The third possibility is a smaller than normal cytoplasmic pool of DA in PKO rats accompanied by a larger vesicular DA pool. This scenario is supported by lower locomotor response of the PKO rats than the response of the WT rats to 2 mg/kg methamphetamine. Methamphetamine dose dependently releases cytoplasmic DA and, at higher doses, vesicular DA through the reversed DAT.

Studies in young PKO mice reported normal DAT function^62,63,65^, increased extracellular DA levels^62^ and a reduction^11,65^ or no change^15,66^ in electrical stimulation-evoked release of DA. An explanation for these seemingly contradictory results was offered by the results from the study of Rial and colleagues^15^: a leakage of DA via the DAT, independent of vesicular release of DA and of DA uptake. Compared to the WT controls, we found unaltered 3-MT/DA and 3-MT/DOPAC ratios in the striatum of parkin knockouts, suggesting that extracellular levels of DA and its metabolism do not differ between the WT and PKO rats. In agreement with this conclusion and with a previous study in 3 month-old male mice^63^, we found unchanged levels of the DAT, a transporter responsible for the rapid reuptake of released DA^67^, in majority of our PKO rats.

Collectively, the data suggests normal DA synthesis, metabolism, release, and uptake, with potentially higher DA content in the storage vesicles and lower DA cytoplasmic pool in PKO rats. The discrepancy in the results from different laboratories might be explained by strain and species differences, differences in generating parkin knockouts (targeting exon 2, 3, or 7 in mice and exon 4 in rats) as well as by different ages of laboratory animals.

ß-PEA behaves as amphetamines and can reverse the DAT, resulting in DA release^68,69^. However, the available data indicates that the levels of ß-PEA have to increase several fold for this process to occur^37^. The finding of only 32% increase in this trace amine levels together with the data discussed above indicate a lack of DA leakage *via* the DAT and also a lack of DA uptake inhibition due to competition of ß-PEA for the binding to the transporter. Of note, the DA-depleting effect of ß-PEA on the VMAT2^68^ could explain previously reported reduction in quantal size of evoked DA release in 2–3 month-old PKO mice^65^, if indeed these mice have increased ß-PEA levels. A potential ß-PEA-induced leakage of DA from the DA storage vesicles could increase formation of toxic products of DA oxidation. We did not detect DA quinones or 4-HNE-protein adducts in the striata of PKO rats. These species were either disposed of by upregulated antioxidant machinery in the PKO rats, including reduced glutathione^60,61,63^, or the hypothesized DA leakage does not take place in the PKO rats. As noted previously, the PKO rats may have a decreased pool of cytoplasmic DA and an increased pool of stored DA compared to WT animals.

In human brain, MAO-B metabolizes ß-PEA and to a high degree DA^70^. In rat brain, MAO-B preferentially metabolizes ß-PEA, while DA is metabolized to a much greater extent, by MAO-A^28–32^. DAergic terminals contain MAO-A while astrocytes contain both MAO-A and MAO-B^71–73^. Our finding of decreased MAO-B activity in striatal mitochondria from the PKO rats indicated functional deficit in this enzyme and reduction in metabolism of ß-PEA in striatal astrocytes. We hypothesized that parkin deficit decreased *MAOB* gene expression. The expression of *MAOB* gene is regulated by peroxisome proliferator-activated receptor gamma coactivator 1-alpha (PGC-1α)^74^. PGC-1α is downregulated by increased levels of PARIS, which is a substrate of parkin; consequently, PARIS content increases in response to parkin protein loss^12^. The decrease in MAO-B activity; however, was not accompanied by decreased expression of *MAOB* gene in the knockouts. Moreover, using SDS-PAGE and western blotting, we found MAO-B protein levels to be unchanged. Our findings are not in agreement with previous findings. Thus, MAO-B expression was increased in B lymphocyte cell line from male PD patients with homozygous deletion of parkin exon 4^33^, while MAO-B activity was increased in whole brain preparations from 1 month-old male mice with homogenous deletion of parkin exon 3^34^ and in the substantia nigra *pars compacta* of PD patients^75^. Conversely, MAO-B (and MAO-A) expression and activity was decreased in cultured DAergic neuroblastoma SH-5YSY cells and non-DAergic cell lines upon overexpression of parkin^33,76^. Of note, MAO-B activity was unchanged in the striatum in female 14 month-old mice with homogenous deletion of parkin exon 3^60^. Our finding of unchanged MAO-B levels, accompanied by the decreased *V*_max_ in parkin knockouts may reflect noncompetitive or uncompetitive inhibition, altered conformation, or different splice variant of MAO-B enzyme with different kinetic characteristics than the one observed in WT rats.

In contrast to rats, MAO-B plays larger role in metabolizing DA in humans and some other species^28,77,78^. Moreover, MAO-A and MAO-B metabolize DA to different degrees in different brain areas^79,80^. Therefore, the discrepancy between the aforementioned and our results can be due to species, gender, and/or regional differences as well as differences in experimental conditions (*in vitro vs*. *in vivo*, measurement of MAO activity *vs*. levels) and generation of PKO phenotype. Compensatory mechanisms could also contribute to the discrepancy. It has been well established that age accounts for different responses in parkin-null rodents and that parkin knockout models compensate better for the lack of parkin protein than parkin mutant models^8^. Moreover, activity of MAO-B in the striatum is decreased in mouse model of pre-symptomatic PD (~59% loss of DAergic axons) and returns to the control levels during symptomatic stage, whereas activity of MAO-A is unchanged in the pre-symptomatic PD model and augmented in the symptomatic PD^13^. Our data is consistent with these findings, suggesting that the 2 month-old male PKO rats may represent a very early stage of PD, manifested by deficit in MAO-B function and increased ß-PEA levels, at which the nigrostriatal system still is able to compensate for consequences of parkin loss of function. Interestingly, REM sleep depravation in rats is associated with reduced activity of MAO-B and increased ß-PEA levels in the striatum^81^. Abnormal REM sleep is also associated with a risk for developing PD^82^ and is a feature of early stages of the disease^83^. Furthermore, REM sleep depravation can be induced by activation of TAAR-1 receptor^84^.

ß-PEA is a ligand for the TAAR-1 receptor; therefore, the decrease in TAAR-1 levels observed in the PKO rats can be an adaptive response to the increase in ß-PEA levels, resulting in attenuated TAAR-1 signaling. The TAAR-1 receptor serves as a stabilizer of DAergic neurotransmission^37^. Thus, if necessary, TAAR-1 increases DAergic neurotransmission *via* phosphorylation-mediated interaction with the DAT and inhibits it *via* upregulation of D2S-mediated DA uptake and *via* downregulation of D2L-mediated postsynaptic signaling. As noted previously, a several fold increase in ß-PEA levels is needed to release DA *via* reversal of the DAT^18,68,69,85^. Consequently, the small alterations in trace aminergic system likely affected D2 receptor signaling only because it does not require high increases in ß-PEA to be regulated. Along these lines, it is plausible that the D2S levels increased while the levels of D2L decreased in the striatum of PKO rats in response to the decrease in TAAR-1 levels, in order to normalize DAergic neurotransmission. The D2L receptor levels may have decreased also in response to constitutively increased ß-PEA levels as ß-PEA was reported to exert a direct stimulating action on postsynaptic DA receptors^42^. Of note, the studies in young PKO mice did not detect changes in total D2 receptor levels^65,86^ or binding for D2/D3 receptors^87^. These results agree with our finding of unchanged immunoreactivity of D2(S + L).

Neither the decrease in D2L levels nor the decrease in the TAAR-1 levels was accompanied by a similar decrease in the gene expression. Increased expression of *DRD2* gene together with decreased levels of D2L protein may reflect impaired translation of *DRD2* mRNA. Alternatively, as changes were detected in the glycosylated forms of D2L and TAAR-1, parkin deficit may trigger changes in posttranslational glycosylation of both receptors in the Golgi apparatus where parkin localizes^88^. As aforementioned, mRNA translation process in parkin knockouts can be potentially decreased; however, not due to altered TDP-43 levels. TDP-43 acts as a repressor of local translation in dendrites and synapses^89,90^ and, therefore, an increase and not a decrease in its levels would result in decreased translation. On the other hand, a mutant TDP-43 protein inhibits protein transport between the endoplasmic reticulum and Golgi apparatus in neuronal cells^91^, suggesting that a deficit in TDP-43 may result in decreased levels of glycosylated D2L and TAAR-1. The identity of the TDP-43-positive 70 kDa band observed in WT and PKO rats, as well as the identity of the ~60 kDa band, detected in WT mice^52,59^, remains to be determined. Our finding is in contrast to the finding of Hebron and colleagues, who found that PKO mice have increased levels of TDP-43 (total and phosphorylated) compared to controls^51^, supporting the notion of species differences between parkin knockout models.

The alterations in ß-PEA, TAAR-1 and D2L receptor levels did not significantly affect basal locomotor activity of the PKO rats. The finding of normal basal locomotion in drug-naïve 2 month-old PKO rats is in agreement with a previous study in 4–8 month old PKO rats^9^ and young adult PKO mice^62^, and suggests normal DAergic neurotransmission in the striatum as well as in the nucleus accumbens, the main brain area mediating horizontal locomotor activity in parkin-deficient rodents. Interestingly, we found a significant decrease in small stereotypic movements (e.g. scratching, grooming) in the PKO rats compared to their WT counterparts, suggesting some dysregulation of the dorsal striatum (e.g. imbalanced D2 and D1 receptors) and cerebellum, two of the brain regions mediating these movements^92,93^. In the study of Dave and colleagues, PKO rats at 4, 6, and 8 months of age did not show any impairment in motor behaviors, including basal locomotion and stereotypic behaviors^9^. Thus, by the 4^th^ month of age, PKO rats apparently undergo adaptive changes to compensate for these small motor impairments.

Amphetamine-induced DA release, an index of newly synthesized cytoplasmic DA, triggers locomotor activation in rodents. Decreased locomotor response to 2 mg/kg methamphetamine in the PKO rats as compared to WT controls supports our hypothesis of smaller cytoplasmic DA pool in the knockouts. Attenuated responses to amphetamines were also noted in PKO mice. Thus, 1–5 mg/kg amphetamine elicited decreased locomotor response in 6 month-old PKO mice^19^ whereas 0.3 mg/kg methamphetamine elicited decreased locomotor response in 12 month-old PKO mice^64^. Another study showed no difference in locomotor response in 3-month-old knockout mice compared to controls after treatment with 4 mg/kg of amphetamine^57^. Although these results are somewhat inconsistent, they suggest that the absence of parkin plays a role in evoked DA neurotransmission *via* decreasing cytoplasmic DA pool. Similarly to PKO rodents, TAAR1 KO mice show normal locomotion at basal conditions but when challenged by amphetamine (2.5 mg/kg), they are hyperactive. This sensitivity has been shown to be due to an elevated spontaneous firing rate of DAergic neurons in the ventral tegmental area^94^. D2L receptor knockout mice display increased basal locomotor activity^95^. Thus, a decreased locomotor response to methamphetamine in the PKO rats may be due to altered activity of the basal ganglia.

As opposed to conditional knockout, compensatory mechanisms occur during traditional transgenesis and, therefore, may be responsible, at least in part, for the lack of major dysregulation, or neurodegeneration, of DAergic system in PKO rodents. Alternatively, the knockout of *Park2* gene does not recapitulate any PD-related phenotype. The PKO rodents; however, display some DAergic system impairments, particularly after exposure to stressors such as methamphetamine, which can oxidatively damage striatal proteins, including parkin^96^. Chronic methamphetamine users have been shown to be at risk for developing PD^97,98^; therefore, our data may be of relevance to this finding. We present some neuronal adaptations that took place in the striatum of male PKO rats until the age of 2 months. It remains to be determined whether these rats represent an early model of PD with the nigrostriatal DA system still being able to compensate for parkin loss of function under non-stressful conditions. In addition, we present species differences in striatal responses to *Park2* knockout that may be important for individualized treatment of PD patients.

## Methods

### Animals

The study was conducted on male 2 month-old Long Evans PKO rats (SAGE Labs, Missouri, MO) and age-matched male WT Long Evans rats (Harlan, Indianapolis, IN) weighing between 250–350 g. Upon arrival, the animals were maintained under standard environmental conditions in the institutional animal facilities in a temperature and humidity controlled room, under a 12 h light/dark cycle with *ad libitum* access to food and water. Animals were weighted every day until the day of sacrifice (~PND63). All animal procedures were approved by the Institutional Animal Care and Use committee at Wayne State University and methods were carried out in accordance with the approved guidelines (A 05–07–13). The description of animal procedures meet the ARRIVE recommended guidelines described by the National Centre for the Replacement, Refinement and Reduction of Animals in Research.

### Tissue collection

PKO and WT Long Evans rat littermates were sacrificed for tissue harvest on the same day using decapitation. Whole striata and cerebella were dissected out, immediately frozen on dry ice, and stored at −80 °C until assayed.

### High performance liquid chromatography

Striatal brain samples were prepared as previously described^99^. The tissue was sonicated in 0.5 mL of cold 0.3 M perchloric acid for 20–30 seconds (with 20% power) and centrifuged at 12, 000 × g for 30 min at 4 °C. The supernatant was collected with great caution (to avoid pellet) in 200 μL high performance liquid chromatography (HPLC) tube. The pellet was re-suspended in 1 M NaOH overnight at 4 °C and protein concentrations were determined using BCA Assay Kit (Thermo Fischer, Waltham, MA). To measure neurotransmitters and their metabolites, 20 or 100 µL of the supernatant was injected into the MDTM mobile phase (Thermo Scientific), at a flow rate of 0.5 mL/min. The samples were separated using C- 18 reverse-phase column (150 × 3.2, 3 µM particle size, Thermo Scientific). The amount of DA, 5-HT and their metabolites: DOPAC, HVA, 3-MT and 5-HIAA were then analyzed based on electrochemical detection and the final values were presented as ng/μg protein.

### Liquid chromatography coupled with tandem mass spectrometry assay of ß-phenylethylamine in rat brain tissue

The concentrations of ß-PEA in rat brain tissue samples were determined by a validated high-performance liquid chromatography coupled with tandem mass spectrometry (LC-MS/MS) method, as described below. Frozen brain tissue samples (weight ranging from 25 to 55 mg) were thawed on ice and homogenized in 10 volumes of ice-cold distilled water by using a Precellys 24 homogenizer (Bertin Technologies, Rockville, MD). The homogenate was transferred to a new Eppendorf tube and 3 volumes of ice-cold methanol were added. The mixture was vortex-mixed for 1 min, and centrifuged at 4 °C, 14,000 rpm, for 20 min. The supernatant was transferred into a new tube and dried under vacuum at 10 °C using a Labconco refrigerated CentriVap concentrator (Kansas city, MO, USA). The residue was reconstituted in 47.5 µL mobile phase consisting of 0.1% formic acid in water and 0.1% formic acid in acetonitrile (98:2, v/v), to which 2.5 µL of the stable isotope-labeled internal standard, 2-phenyl-d5-ethylamine (d5-PEA), was added. The reconstitution solution was centrifuged at 4 °C, 14,000 RPM, for 5 min. Five microliter of the supernatant was subjected to LC-MS/MS analysis using a SHIMADZU (Kyoto, Japan) Nexera ultra high performance liquid chromatography system coupled with an AB SCIEX (Foster City, CA) QTRAP 6500 mass spectrometer.

Chromatographic separation was achieved on a Synergy polar-RP column (2 × 100 mm, 4 µm, 80 Å) (Phenomenex, Torrance, CA, USA) at 35 °C with a gradient mobile phase A (0.1% formic acid in double deionized water) and B (0.1% formic acid in acetonitrile), at a flow rate of 0.3 mL/min. The optimized gradient program (shown as time, mobile phase B) was as follows: 0–0.5 min, 0 to 2%; 0.5–1.5 min, 2% to 20%; 1.5–5 min, 20% to 65%; 5–6.5 min, 65%; 6.5–7 min, 65% to 100%; 7–8.5 min, 100%; 8.5–8.7 min, 0%; and 8.7–12 min, 0%. The autosampler was set at 4 °C. The column effluent was monitored using the QTRAP 6500 mass spectrometer equipped with electrospray ionization source. The optimal MS conditions were obtained by direct infusion of the standard solution of ß-PEA and d5-PEA into the ion source. ß-PEA and d5-PEA were monitored under positive ionization mode at the MS transitions (m/z) of 122.0 > 105.0 and 127.0 > 110.0, respectively. The Turbo ion-spray voltage was 5500 V and the temperature was set at 475 °C. Collision gas was optimized at medium level, and curtain gas was set at 30 psi with both ion source gas 1 and 2 at 50 psi. The declustering potential was set at 21 and 25 V for ß-PEA and d5-PEA, respectively. The collision energy was 16 and 17 V for PEA and d5-PEA, respectively. The calibration curve of PEA was constructed at the concentration range of 0.2 to 100 nM. Intra- and inter-day precision and accuracy of the calibrators and quality control samples were within the generally acceptable bioanalytical criteria (<15%).

### SDS-PAGE and western blotting

 Tissue lysates and synaptosomal fractions (synaptosomal, membrane, and vesicular fractions) were prepared from striatal and cerebellar brain samples by differential centrifugation as previously described (Liu *et al*.^100^). Tissue pieces were homogenized in ice-cold RIPA buffer or in 0.5 mL 0.32 M sucrose solution containing protease inhibitor cocktail and centrifuged at 10,000 × g for 15 min or at 800 × *g* for 24 min, respectively, at 4 °C. The supernatant form the low-speed centrifugation was then centrifuged again at 22,000 × *g* for 17 min at 4 °C, and the pellet dissolved in 150 µL ice-cold distilled water was retained as synaptosomal fraction. Part of the synaptosomal fraction was further centrifuged at 22,000 × *g* for 17 min at 4 °C and the supernatant was retained as cytosolic fraction while the re-suspended pellet dissolved in ice-cold distilled water was retained as a membrane fraction. Sample protein concentrations were determined using BCA Assay Kit (Thermo Fischer). Striatal and cerebellar samples were diluted to even protein concentrations in LDS sample buffer (Thermo Fischer) containing 5% ß-mercaptoethanol (Thermo Fischer). The samples were subsequently heated at 70 °C for 10 min, loaded onto sodium dodecyl sulfate-polyacrylamide gel (4–12% SDS-PAGE) (20–30 μg of protein per lane) and run at a constant voltage of 100 V for 60 min. Proteins were transferred onto methanol-activated PVDF membranes at 0.4 A for 60 min. Transfer efficiency and protein loading was asessed using Ponceau S dye (Sigma-Aldrich, St. Louis, MO). The membranes were then blocked for 60 min at room temperature in 5% non-fat dry milk dissolved in TBST (50 mM Tris-HCl pH 7.6, 150 mM NaCl, 0.05% Triton X-100). Membranes were then incubated overnight at 4 °C in one of the following primary antibodies diluted in the blocking buffer; anti-VMAT2 (1:3000, NB110-68123, Novus Biologicals, Littleton, CO), anti-DAT (1:500, SC1433, Santa Cruz Biotechnology, Dallas, TX), anti-parkin (1:1000, mouse 4211 S or rabbit 2132, Cell Signaling Technology, Danvers, MA), anti-TH (1:1000, 2043879, EMD Millipore, Billerica, MA,), anti-β-actin (1:2000, 3700, Cell Signaling Technology), anti-TAAR-1 (1:1000, sc398096, Santa Cruz Biotechnology), anti-TDP-43 (1:1000, 1078-2-AP, Proteintech, Chicago, IL), anti-MAO-B (1:1000, M1946, Sigma-Aldrich), anti D2L (1:1000, AB1792P, EDM Millipore), anti D2(S+L) (1:1000, AB1558, EDM Milliore) antibody, or antibody against 4-HNE-protein conjugates (1 µg/mL, R&D Systems, Minneapolis, MN). Membranes were washed 3 times for 5 minutes in TBST and incubated with appropriate secondary antibodies (anti-rabbit, anti-goat, or anti-mouse, 1:10,000) for 60 min at room temperature. The membranes were washed again 3 times in TBST, incubated for 2 min with SuperSignal West Pico chemiluminescence substrate (Thermo Scientific), and visualized using ECL detection and LAS4000 Bioimager (GE Healthcare, Piscataway, NJ). The levels of DA quinone-conjugated proteins in striatal tissue were measured by redox-cycling staining according to the previously published protocol^101^. Briefly, the membrane with striatal proteins, separated by SDS-PAGE, were stained with Ponceau S (0.1% in 5% acetic acid), washed with water, and immersed in a solution of 0.24 mM nitro blue tetrazolium, 2 M potassium glycinate (pH 10.0) for 45 min in the dark. The reaction was stopped by immersing the membrane in the borate buffer (0.1 M, pH 10.0). DA quinone-bound bovine serum albumin (BSA) was used as a positive control. All immunoreactivities were quantified using ImageJ (National Institutes of Health, Bethesda, MD) and presented as a relative optical density units per mg proteins.

### Immunohistochemistry

Fixed brain tissues were processed as previously described^100^. Citrate buffer antigen retrieval (ThermoFisher, Waltham, MA, USA) was applied to all tissue sections. The sections were incubated overnight at 4 °C with an anti-TAAR-1 antibody (sc398096, Santa Cruz Biotechnology) diluted 1:100 in the blocking buffer. The sections were then incubated for 2.5 h at room temperature with Alexa Fluor-488 conjugated secondary antibody (Invitrogen, Carlsbad, CA, USA). DRAQ5 (Invitrogen) was used to stain nuclei. The sections were then mounted using Fluoromount mounting medium (Southern Biotech, Birmingham, AL, USA). The immunostaining on each slice (3 sections per slice) was imaged using the Leica TCS SPE-II laser scanning confocal microscope (Leica, Wetzlar, Germany) and averaged per rat.

### Monoamine oxidase activity assay

MAO-A and MAO-B activity in striatal mitochondria was analyzed based on fluorimetric technique using MAO Assay Kit (BioAssay systems) and Gen 5 All-in-one microplate reader software. Striatal mitochondria were isolated using commercially available Mitochondrial Isolation Kit for Tissue (Thermo Scientific, Rockford, IL). Striatal tissue were homogenized in 800 µL of bovine serum albumin (BSA)/Reagent A solution and dissolved in 800 µL Reagent C and centrifuged at 800 × g for 10 min at 4 °C. The supernatant was further centrifuged at 12,000 × *g* for 15 min at 4 °C and the mitochondrial pellet surface was washed with 500 µL wash buffer and dissolved with 1% digitonin, protease inhibitor in Reagent C. The re-suspended pellet was sonicated at 20% power, three times for 5 min, and centrifuged. The supernatants were used as sources for MAO. BCA assay was used for protein estimation. In order to determine MAO-A and MAO-B activities, samples were incubated with the substrate, p-tyramine, at 1 mM following establishing controls with 0.5 µM MAO-A inhibitor, clorgyline or 0.5 µM MAO-B inhibitor, pargyline, respectively. Each MAO sample of 45 µL was transferred into four separate wells of dark 96-well plate (BD Bioscience, Franklin Lakes, NJ), together with 5 µL of assay buffer, 5 µL of 10 µM clorgyline, or 5 µL of 10 µM pargyline. The solutions were then incubated for 10 min at room temperature for the inhibitors to block the reaction. To each well 47 µL assay buffer, 1 µL p-tyramine, 1 µL dye reagent, and 1 µL horse radish peroxidase (HRP) enzyme) was added and solutions were incubated for 20 min in the dark. The relative fluorescence intensity was measured at λ_ext_ = 530/λ_Em_ = 585 nm), at room temperature (25 °C) for 60 minutes using Synergy H1 Hybrid reader (BioTek Highland Pak, VT).

### Real-Time Polymerase Chain Reaction

The striata were disrupted with tissue grinder (Wheaton) and homogenized with a 20-gauge needle (BD). Total RNAs was extracted from the homogenates using RNeasy Mini Kit (Qiagen) in a presence of RNase-Free DNase Set (Qiagen) to remove genomic DNA. Total RNA concentration was measured using NanoDrop Lite Spectrophotometer (Thermo Scientific). One-step quantitative PCR was performed with Brilliant III ultra-fast SYBR® green qRT-PCR master mix kit (Agilent Technologies, Santa Clara, CA) using a Stratagene Mx3005 P system (Agilent Technologies). Each reaction started with 100 ng RNA which was amplified by 40 cycles of 95 °C for 20 sec and 60 °C for 20 sec with an initial step of 55 °C for 10 min and 95 °C for 3 min. Cycle threshold (Ct) values were determined by MxPro software (Agilent Technologies). Gene expressions were normalized by the expression of GAPDH and calculated using delta CT method. The primer sets (Qiagen) were the following: (i) *TAAR1* (Cat. # QT00412069), *MAOB* (Cat. # QT00180887), *DRD2* (Cat. # QT01081990), *CHRNA7* (Cat. # QT00176561), and *GAPDH* (Cat. # QT00199633).

### Drug treatment and motor activity measurement

Separate groups of PKO and WT rats were intraperitoneally (i.p.) injected with 2 mg/kg (free-base) of *d*-methamphetamine hydrochloride (Sigma-Aldrich, St. Louis, MO, USA) dissolved in sterile saline (0.9% NaCl). The gross motor activity of all the animals was assessed using the Opto-Varimex 4 AutoTrack open-field Plexiglass chamber (Columbus Instruments, Columbus, OH). At the beginning of each session, the animals were placed in the middle of the Plexiglass chamber, and their subsequent movement and position were recorded continuously. For each drug-naïve animal, motor activity was assessed during 30-min session. Activity of methamphetamine-treated rats was recorded for 100 min. Horizontal locomotor activity (distance travelled), and stereotypic movements (time spent) were calculated automatically from the raw beam-break data by the Opto-Varimex software.

### Statistical analysis

The comparisons made in the study were pre-planned comparisons. Data sets were analyzed using two-tailed unpaired Student’s t-test or two-way ANOVA followed by Holm-Sidak’s *post hoc* test. Statistical analyses were performed using GraphPad Prism program (GraphPad Software, San Diego, CA) on the raw data with the exception of the western blotting data. The western blotting data are expressed as relative optical density units on each gel normalized to controls. This approach normalized differences across blots, allowing for standardization across the treatment groups. The data are presented as mean ± standard error (SEM). Statistical significance was set at p < 0.05.

## Electronic supplementary material

Supplementary Information
