# Supplementary material for: Characterization of Dopaminergic System in the Striatum of Young Adult Park2−/− Knockout Rats
Source: Sci Rep. 2018 Jan 24;8:1517. doi: 10.1038/s41598-017-18526-0 (PMC5784013; doi:10.1038/s41598-017-18526-0)
Supplement: Supplementary file 1 — Supplementary Information [file 41598_2017_18526_MOESM1_ESM.pdf]

Supplementary Information for

Characterization of Dopaminergic System in the Striatum of Young Adult *Park2*$^{-/-}$ Knockout Rats

Jickssa M. Gemechu[1,2], Akhil Sharma[1], Dongyue Yu[1], Yuran Xie [1,4], Olivia M. Merkel[1,5], and Anna Moszczynska[1]

[1] Department of Pharmaceutical Sciences, Wayne State University, Detroit, MI, USA

[2] Department of Biomedical Sciences, OUWB School of Medicine, Rochester, MI, USA

[3] Van Andel Research Institute, Grand Rapids, MI, USA

[4] Boston Biomedical Inc., Allston, MA, USA

[5] Department of Pharmacy, Ludwig-Maximilians University of Munich, Munich, Germany

Corresponding author:

Anna Moszczynska, Ph.D., Department of Pharmaceutical Sciences, Wayne State University, 259 Mack Ave., Detroit MI 48202, amosz@wayne.edu

This file include (full-length blots):

1. Supplementary figure 1A
2. Supplementary figure 2A, 2Bi and 2Bii
3. Supplementary figure 3B and C
4. Supplementary figure 5B and C
5. Supplementary figure 6A and B
6. Supplementary figure 8C

1A

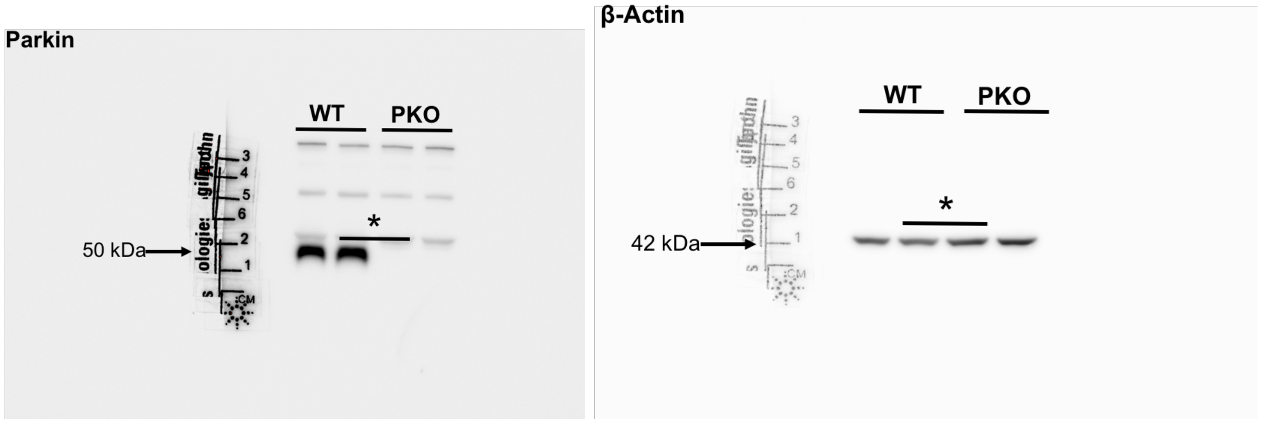

Anti-parkin antibody Cat #4211

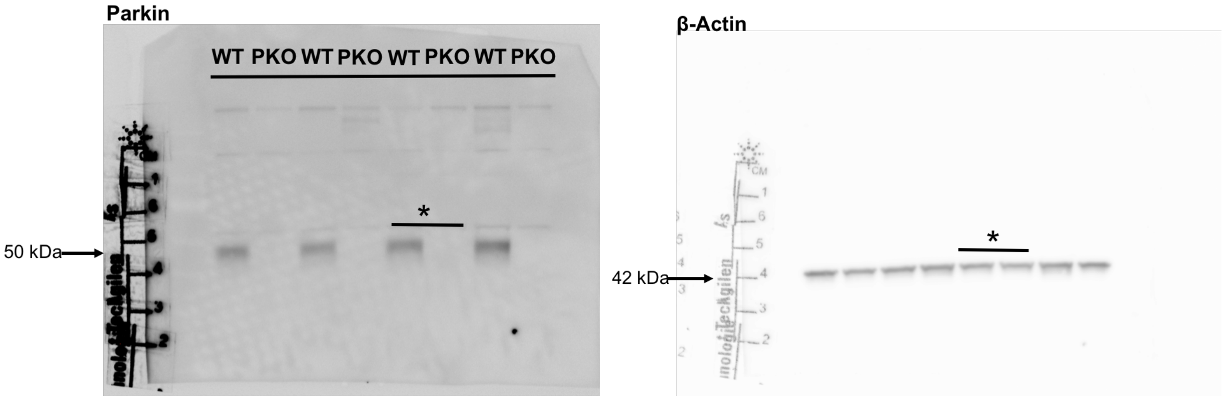

Anti-parkin antibody Cat #2132

Supplementary Figure 1A. Full-length blots demonstrating a complete loss of parkin immunoreactivity in the parkin knockout (PKO) as compared to the wild type (WT) Long Evans male 2 month-old rats (n=6). The striata were dissected out and analyzed for parkin immunoreactivity using SDS-PAGE and western blot analysis with two different anti-parkin antibodies. Asterisks mark the blots presented in the manuscript. Abbreviations: PKO, parkin knockout; WT, wild type.

2A

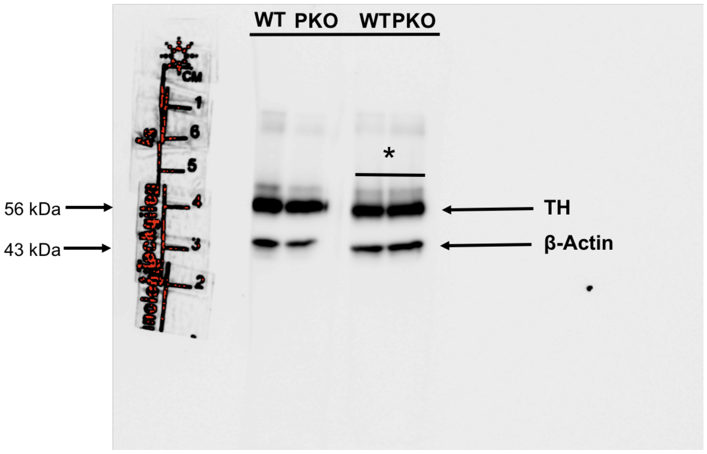

2Bi

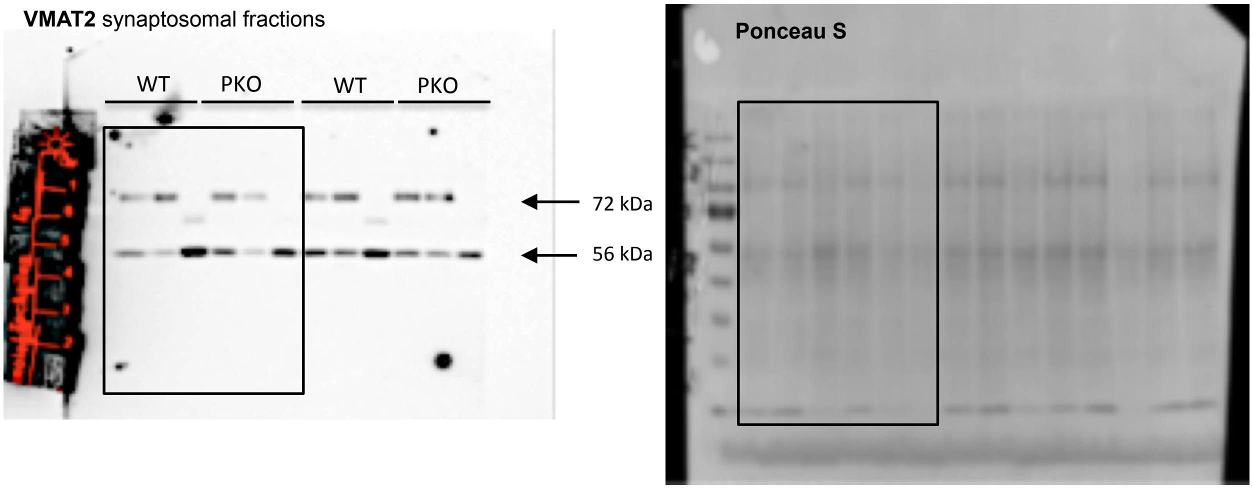

2Bii

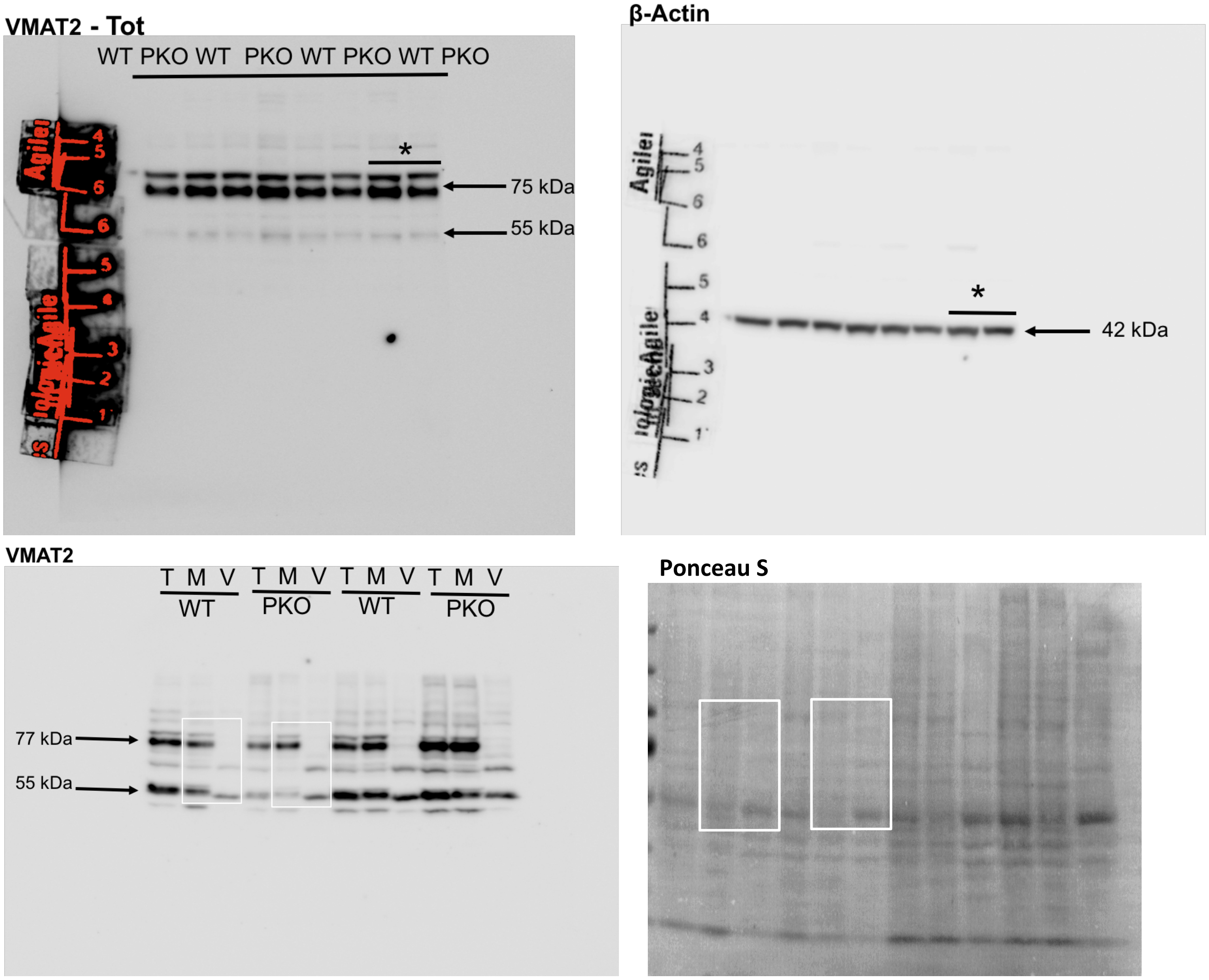

Supplementary Figure 2. Full blots demonstrating the effect of parkin protein loss on synthesis and storage of striatal dopamine. Whole synaptosomes and synaptosomal fractions were isolated form striata of the 2 month-old parkin knockout (PKO) and wild type (WT) Long Evans male rats. **(A)** Tyrosine hydroxylase (TH) band and ß-actin band obtained by double labeling of the same membrane. **(Bi)** VMAT2 protein in synaptosomal fractions (T, M, V, T, M, V etc.) from the striata of WT and PKO rats with corresponding protein content assessed by Ponceau S staining. **(Bii)** Top: full blot showing VMAT2 levels in total synaptosomal fractions in WT and

PKO rats and corresponding ß-actin levels. Bottom: another blot with VMAT2 protein in synaptosomal fractions from the striata of WT and PKO rats with corresponding protein content assessed by Ponceau S staining. In this blot, VMAT2 levels in the vesicular fraction are presented better than in the Bi blot. Asterisks or boxes mark the blots presented in the manuscript. Additional two lanes (from the right) in Ponceau S blots are for another unrelated staining. Abbreviations: Tot (T), total synaptosomal fraction; Mem (M), membrane fraction; Ves (V), vesicular fraction; VMAT2, vesicular monoamine transporter.

3B

MAO-B **ß-Actin**

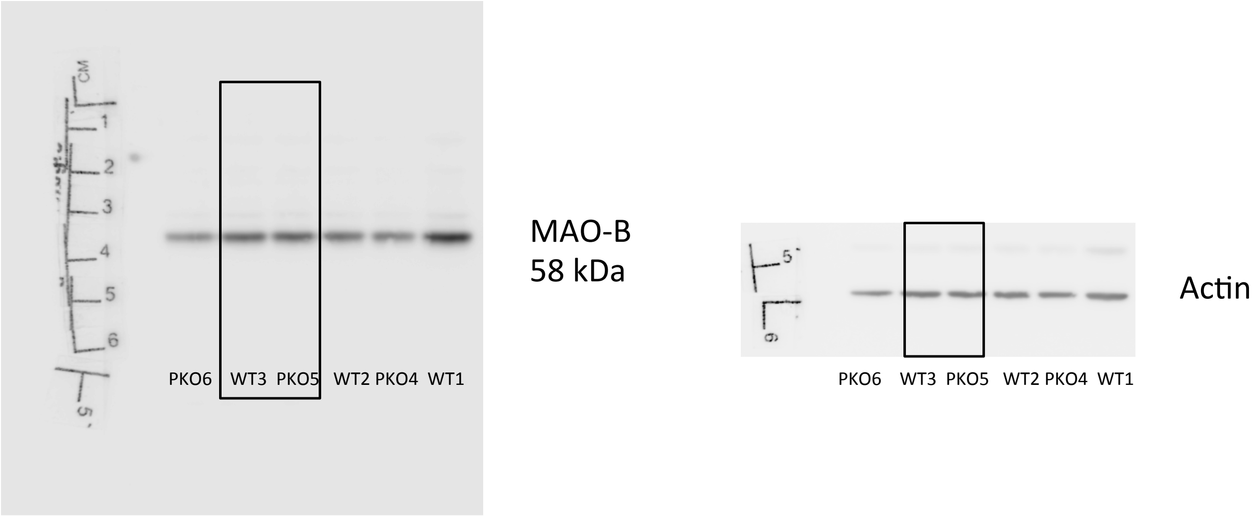

3C

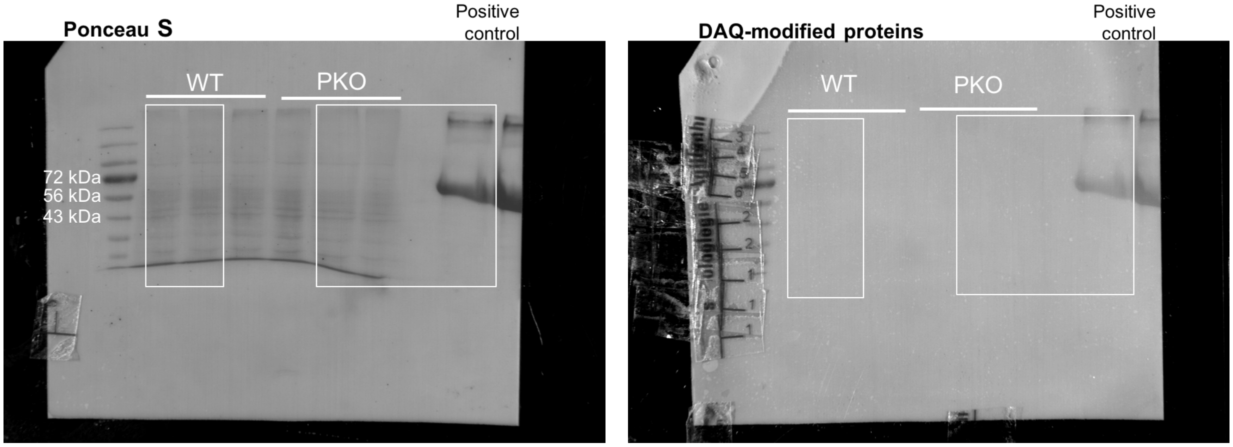

Supplementary Figure 3. (B) Full blot showing monoamine oxidase B (MAO-B) labeling of striatal lysates from wild type (WT) and parkin knockout (PKO) rats with the corresponding blot showing ß-actin labeling. WT designate wild type samples whereas numbers designate parkin knockout samples from left (L) or right (R) hemisphere. **(C)** Full length blots showing lack of immunoreactivity for proteins modified by DA quinones in the striatal lysates of WT and PKO rats; DA quinone-modified bovine serum albumin serves as a positive control. Boxes indicate the specific lanes presented in the manuscript. Abbreviations: DAQ, dopamine quinone.

5B

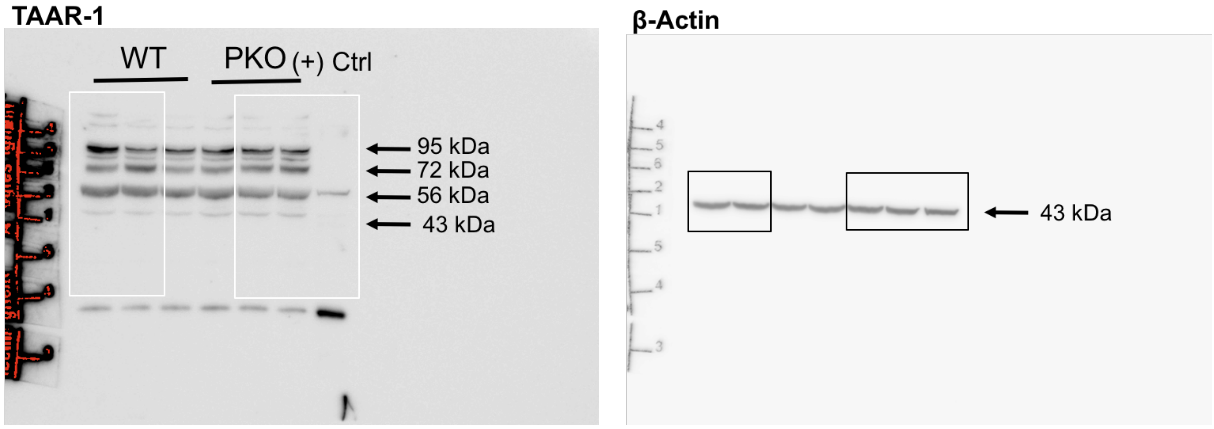

5C

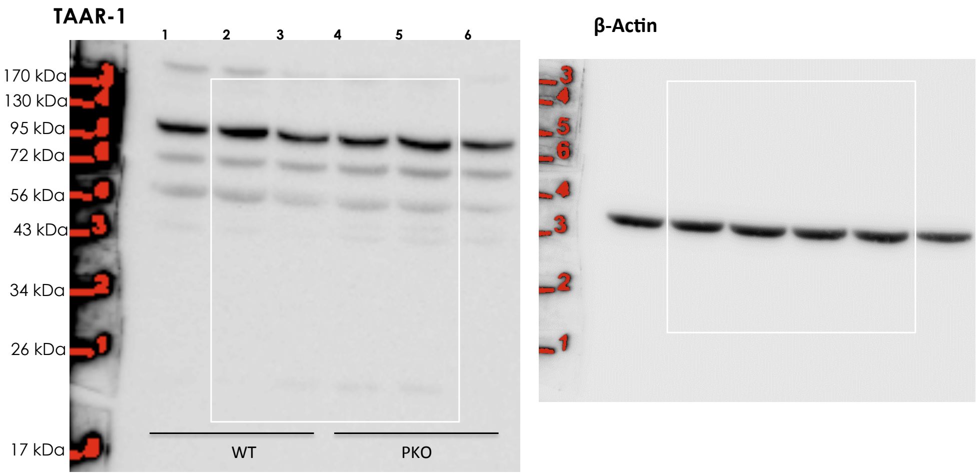

Supplementary Figure 5. (B) Full length blot demonstrating immunoreactivity of trace amine-associated receptor 1 (TAAR-1) in the **(B)** striatum and **(C)** cerebellum of parkin knockout (PKO) and wild type (WT) Long Evans male rats and wild type (WT) controls. On the right are blots with corresponding ß-actin labeling. Boxes indicate the specific lanes presented in the manuscript. (+)Ctrl stands for positive control, which was a lysate rich in TAAR1.

6A

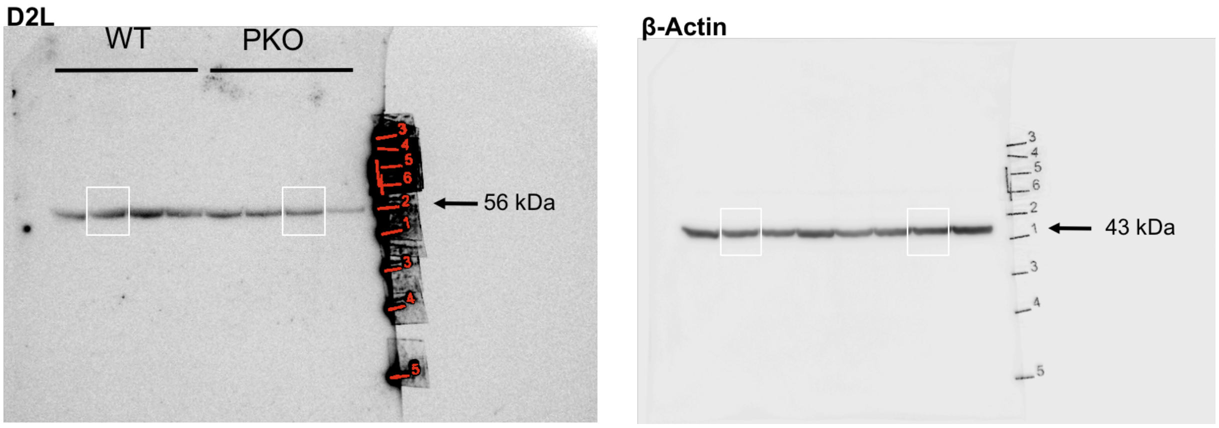

6B

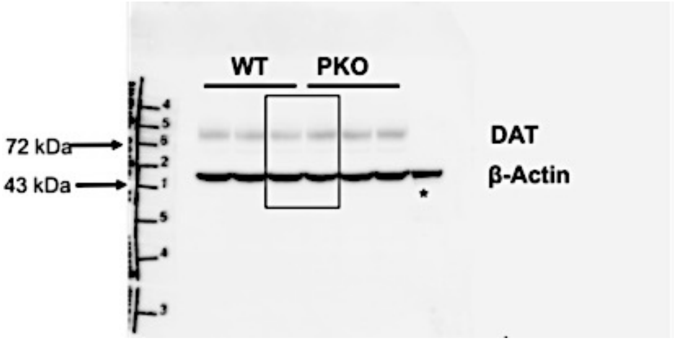

Supplementary Figure 6. Full length blots demonstrating the effect of parkin protein loss on the levels of postsynaptic dopamine D2 receptor (D2L) **(A)** and dopamine transporter (DAT) **(B)** in the striatum in parkin knockout (PKO) as compared to wild type (WT) rats. Tissue lysates were prepared form striata of the 2 month-old male rats. **(A)** The levels of postsynaptic D2 receptor (D2L) (~55 kDa) were significantly decreased in the striatum of PKO rats compared to the striatum of WT controls. **(B)** The immunoreactivity of glycosylated dopamine transporter (DAT) (detected at ~70 kDa) did not significantly differ between the PKO and WT phenotypes in majority of the PKO rats. The specific lanes shown in the manuscript are indicated by boxes. Asterisk indicates negative control - no-primary antibody control.

8C

TDP-43

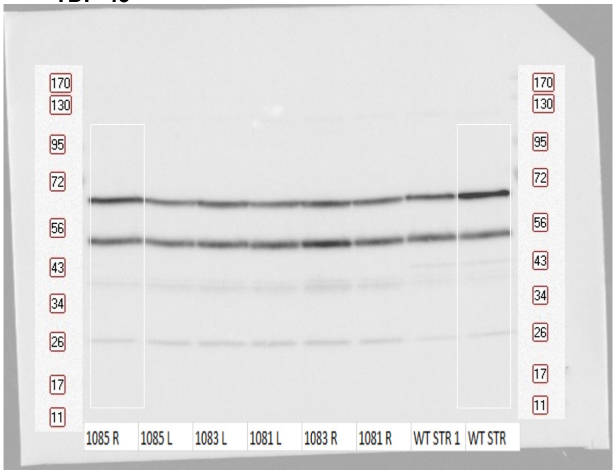

β-Actin

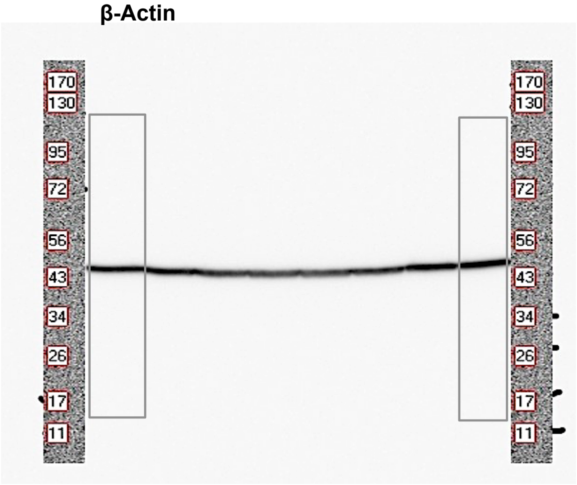

Supplementary Figure 8. (C) Full length blot demonstrating the effects of parkin protein loss on the levels of TDP-43 and the blot with corresponding ß-actin levels. WT designate wild type samples whereas numbers designate different PKO samples. The specific lanes shown in the manuscript are indicated by boxes; they were chosen as to have similar ß-actin levels.
